# Predictive Performance of Radiomics-Based Machine Learning for Colorectal Cancer Recurrence Risk: Systematic Review and Meta-Analysis

**DOI:** 10.2196/78644

**Published:** 2025-11-28

**Authors:** Yuan Sun, Bo Li, Chuanlan Ju, Liming Hu, Huiyi Sun, Jing An, Tae-Hun Kim, Zhijun Bu, Zeyang Shi, Jianping Liu, Zhaolan Liu

**Affiliations:** 1 Centre for Evidence-based Chinese Medicine, Beijing University of Chinese Medicine, No.11 East Beisanhuan Road, Heping Street, Chaoyang District, Beijing, 100029, China, 8613552999260; 2 Department of Public Health, Shandong College of Traditional Chinese Medicine, Yantai, China; 3 Preventive Medicine Association, Yantai Center for Disease Control and Prevention, Yantai, China; 4 Department of Public Health, Yantai Hospital of Traditional Chinese Medicine, Yantai, China; 5 Department of Spleen, Stomach, Liver and Gallbladder, Dongfang Hospital, Beijing University of Chinese Medicine, Beijing, China; 6 Department of Spleen, Stomach, Liver and Gallbladder, Dongzhimen Hospital, Beijing University of Chinese Medicine, Beijing, China; 7 Department of Spleen and Stomach, The Third Affiliated Hospital of Beijing University of Chinese Medicine, Beijing, China; 8 Korean Medicine Clinical Trial Center, Kyung Hee University Korean Medicine Hospital, Seoul, Republic of Korea

**Keywords:** colorectal cancer, radiomics, meta-analysis, clinical prediction model, PRISMA, Preferred Reporting Items for Systematic Reviews and Meta-Analyses

## Abstract

**Background:**

Predicting colorectal cancer (CRC) recurrence risk remains a challenge in clinical practice. Owing to the widespread use of radiomics in CRC diagnosis and treatment, some researchers recently explored the effectiveness of radiomics-based models in forecasting CRC recurrence risk. Nonetheless, the lack of systematic evidence of the efficacy of such models has hampered their clinical adoption.

**Objective:**

This study aimed to explore the value of radiomics in predicting CRC recurrence, providing a scholarly rationale for developing more specific interventions.

**Methods:**

Overall, 4 databases (Embase, PubMed, the Cochrane Library, and Web of Science) were searched for relevant articles from inception to January 1, 2025. We included studies that developed or validated radiomics-based machine learning models for predicting CRC recurrence using computed tomography or magnetic resonance imaging and provided discriminative performance metrics (c-index). Nonoriginal articles, studies that did not develop a model, and those lacking clear outcome measures were excluded from the study. The quality of the included original studies was assessed using the Radiomics Quality Score. A bivariate mixed-effects model was used to conduct a meta-analysis in which the c-index values with 95% CI were pooled. For the meta-analysis, subgroup analyses were conducted separately on the validation and training sets.

**Results:**

This meta-analysis included 17 original studies involving 4600 patients with CRC. The quality of the identified studies was low (mean Radiomics Quality Score 13.23/36, SD 2.56), with limitations in prospective design and biological validation. In the validation set, the c-index values based on clinical features, radiomics features, and radiomics features combined with clinical features were 0.73 (95% CI 0.68‐0.79), 0.80 (95% CI 0.75‐0.85), and 0.83 (95% CI 0.79‐0.87), respectively. In the internal validation set, the c-index values based on clinical features, radiomics features, and radiomics features+clinical features were 0.70 (95% CI 0.61‐0.79), 0.83 (95% CI 0.78‐0.88), and 0.83 (95% CI 0.78‐0.88), respectively. Finally, in the external validation set, the c-index values based on clinical features, radiomics features, and radiomics features combined with clinical features were 0.76 (95% CI 0.70‐0.83), 0.75 (95% CI 0.66‐0.83), and 0.83 (95% CI 0.78‐0.88), respectively.

**Conclusions:**

Radiomics-based machine learning models, especially those integrating radiomics and clinical features, showed promising predictive performance for CRC recurrence risk. However, this study has several limitations, such as moderate study quality, limited sample size, and high heterogeneity in modeling approaches. These findings suggest the potential clinical value of integrated models in risk stratification and their potential to enhance personalized treatment, though further high-quality prospective studies are warranted.

## Introduction

Colorectal cancer (CRC) is ranked as the third most prevalent malignancy and the second most common cause of cancer-related deaths worldwide [1,2]. According to the International Agency for Research on Cancer, there were 1,926,100 (9.6%) new CRC cases and 903,900 (9.3%) CRC-related deaths in 2022 alone [2], highlighting CRC as a major public health concern. Although notable developments have been reported for targeted therapies, adjuvant chemotherapy, as well as radical surgery, cases of tumor recurrence are still a major issue leading to poor survival outcomes [3]. Therefore, early identification of CRC recurrence risk in clinical practice and the development of precise interventions would be imperative for improved clinical outcomes.

Machine learning (ML) and radiomics have recently gained widespread attention in oncological diagnoses and treatments, including in CRC [4,5]. Radiomics is an interdisciplinary technology that quantitatively analyzes high-dimensional features in medical images (eg, computed tomography [CT], magnetic resonance imaging [MRI], and positron emission tomography) to mine pathological information not visible to the naked eye. It entails segmenting regions of interest (ROIs) from medical images, extracting predefined mathematical features, and integrating them with ML or deep learning (DL) algorithms to generate interpretable clinical prediction models [6]. This technique has been extensively documented in the literature for cancer diagnosis and survival prediction [7-9]. Specifically within CRC, several studies have explored radiomics for recurrence prediction [10], using various image modalities including CT and MRI.

However, the available primary studies adopt diverse methodologies, using different imaging protocols, segmentation methods, feature extraction techniques, and model validation approaches. Therefore, the findings from such studies are inconsistent, making the clinical translation of individual models challenging. Although some reviews have summarized the application of radiomics in CRC management [11,12], none have specifically synthesized and quantitatively evaluated the predictive performance of radiomics-based ML models for CRC recurrence risk through meta-analysis. In addition, most of the previous reviews failed to discuss how integrative modeling, combined with radiomic and clinical variables, facilitates clinical decision-making to mitigate recurrence and improve patient management.

In this meta-analysis, we aimed to systematically assess the predictive performance of radiomics-based ML models on the risk of CRC recurrence, focusing on models incorporating both imaging and clinical features. In addition, we explored whether integrated approaches offer superior predictive accuracy compared to models using either data type alone. By comprehensively evaluating the existing evidence and its limitations, this study aims to provide robust evidence that can inform the development of effective, personalized intervention strategies for CRC.

## Methods

### Study Registration

This study was submitted to PROSPERO (International Prospective Register of Systematic Reviews; ID: CRD420250656632) and adhered to the PRISMA (Preferred Reporting Items for Systematic Reviews and Meta-Analyses) guidelines.

### Eligibility Criteria

The inclusion and exclusion criteria for study selection are summarized in Textbox 1.

Textbox 1. **Inclusion and exclusion criteria.**

**Inclusion criteria**

- Studies enrolling patients with colorectal cancer with no requirement for cancer staging and metastatic resections.
- Studies involving radiomics-based machine learning (ML) predictive models and imaging data (magnetic resonance imaging and computed tomography)–based radiomics models.
- Studies published in English.

**Exclusion criteria**

- Meta-analyses, reviews, guidelines, expert opinions, and conference abstracts.
- Studies that performed variance factor analysis but did not construct a grammatical ML model.
- Studies without clearly defined endpoint metrics that could make it difficult to assess ML predictive accuracy.
- Studies that only performed image segmentation without constructing a full model.

### Data Sources and Search Strategy

We systematically searched 4 databases (Web of Science, Cochrane Library, Embase, and PubMed) from inception to 1 January 2025. The search strategy involved a combination of Medical Subject Heading terms and free-text words. Detailed search strategies are provided in Table S1 in Multimedia Appendix 1 [10,13-28]. The search had no regional restrictions.

### Study Selection and Data Extraction

The literature retrieved from the searched databases was imported into EndNote software (Clarivate). After eliminating redundant entries, the remaining articles were systematically screened based on titles and abstracts to discard references that did not meet the predetermined eligibility requirements. The full texts of the remnant studies were screened to obtain additional relevant studies.

Data extracted included titles, year of publication, first author, study type, country, patient sources, study design, treatment regimen, outcome definitions, radiomics source, segmentation method, number of imaging investigators, ROI segmentation software, number of cases and total number of outcome events in the validation and training sets, type of model used, variable screening method, modeling variables, overfitting assessment, and model rating metrics.

Two authors (YS and BL) independently selected the studies and extracted the data, with a third researcher (JA) consulted to resolve any disputes. The interrater agreement between the 2 independent reviewers during the study selection process was excellent, with a Cohen κ value of 0.895.

### Study Quality and Risk-of-Bias Assessment

This meta-analysis included studies that used radiomics-based ML models to predict CRC recurrence risk. The Radiomics Quality Score (RQS) was used to determine the reporting completeness and methodological robustness of the included articles [29]. This 16-item scoring system, with a maximum score of 36, is specifically designed to facilitate radiomics studies that encompass various aspects, including image protocol quality, multiple segmentation processes, phantom studies across all scanners, imaging conducted at multiple time points, feature reduction techniques, and multivariable analyses incorporating nonradiomics features. In addition, it addresses the detection and discussion of biological correlates, cut-off analyses, discrimination statistics, calibration statistics, and prospective studies registered in trial databases. Furthermore, the system emphasizes the importance of validation, comparison against established “gold standards,” potential clinical use, cost-effectiveness analyses, and adherence to principles of open science and data sharing. Existing radiological studies are challenging to evaluate within the RQS framework, as they often lack phantom studies across different scanners, imaging at multiple time points, identification and discussion of biological correlates, prospective trial registration, and cost-effectiveness analyses.

Two investigators (YS and CJ) administered the RQS measure and cross-checked the results upon completion. A third investigator (HS) was consulted to resolve any disputes.

### Synthesis Methods

This meta-analysis aimed to synthesize the discrimination metrics (c-index) for evaluating the overall accuracy of ML models. For primary studies with 95% CI values or SEs for c-index values, the standard errors were estimated using Debray et al [30] methodological framework. Heterogeneity across studies was evaluated quantitatively using the *I*² index. The restricted maximum likelihood method was used to estimate between-group variance, which is recommended for its improved performance in handling heterogeneity, particularly when the number of studies is limited [31]. Given significant heterogeneity (defined as *I²*>50%), a random-effects model was adopted for meta-analysis, and sensitivity analyses were performed. Publication bias was assessed through funnel plot asymmetry analysis and Egger test. Subgroup analyses were conducted to further detect possible sources of heterogeneity. Subgroup analyses were conducted based on imaging modality (CT vs MRI) and dataset type (training vs validation sets). The meta-analysis was conducted in Stata 15 (StataCorp LLC). In addition, to calculate the 95% prediction intervals, which estimate the range within which the true effect of a future study would be expected to fall, we used R software (version 4.4.3; R Development Core Team).

### Quality of the Evidence (Grading of Recommendations, Assessment, Development, and Evaluation Assessment)

The GRADE (Grading of Recommendations, Assessment, Development, and Evaluation) approach was used to determine the overall certainty of evidence for our primary outcome (predictive performance measured by c-index) [32]. Given that the included studies were observational in design, the initial evidence level was low. Therefore, we evaluated the evidence across the 5 GRADE domains (risk of bias [RoB], inconsistency, indirectness, imprecision, and publication bias), creating a final certainty rating for each model comparison.

## Results

### Study Selection

**Figure 1.**
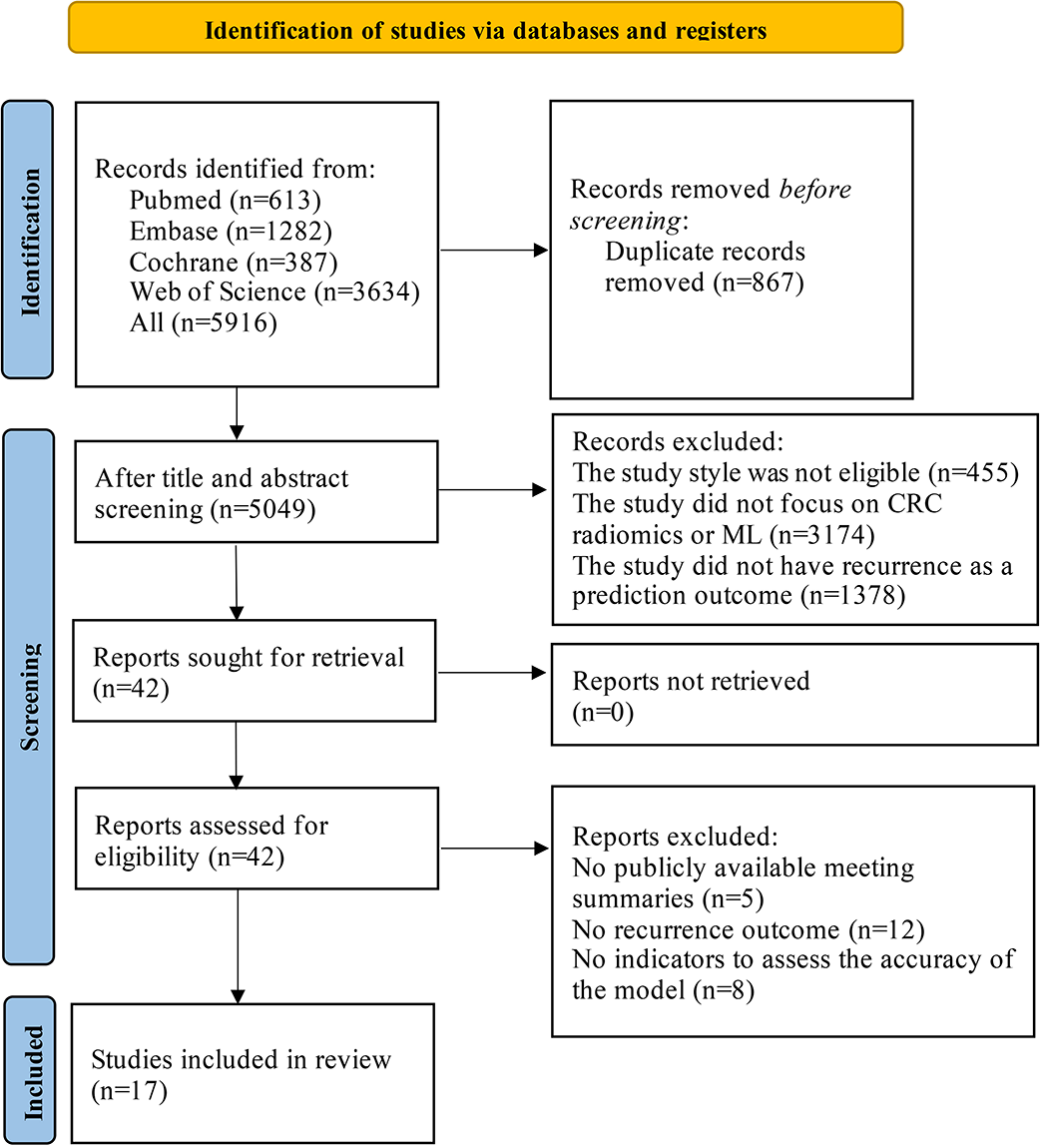
PRISMA (Preferred Reporting Items for Systematic Reviews and Meta-Analyses) flowchart of the study selection procedure for this systematic review and meta-analysis. CRC: colorectal cancer; ML: machine learning.

The initial search of the 4 databases yielded 5916 articles, of which 5049 remained after removing duplicates. Two reviewers independently assessed the title and abstract of all remaining articles, of which 5007 were excluded due to inconsistencies with the study goals. Reasons for exclusion were primarily nonrelevant study type (eg, reviews), not focusing on CRC radiomics or ML, or not having recurrence as a prediction outcome. The remaining articles were further subjected to a careful full-text review, after which 25 articles (5 conference abstracts, 12 articles without CRC recurrence endpoints, and 8 articles missing indicators for assessing model accuracy) were removed. Therefore, only 17 articles [10,13-28] were included in the study’s analyses. Figure 1 shows the study selection process.

### Features of the Included Study

This meta-analysis included 17 studies (involving 4600 patients with CRC) [10,13-28], which were published between 2019 and 2025. All 17 studies were cohort investigations, of which 12 were conducted in China [10,13-15,17,18,20,21,23,25-27], and the rest were conducted in Canada [16], France [24], South Korea [28], the Netherlands [19], and the United States [22]. Furthermore, 8 [16,20,22,23,25-28] and 9 [10,13-15,17-19,21,24] studies were single- and multicenter investigations, respectively. In addition, 7 [16-18,20,23-25], 9 [10,13-15,19,21,22,26,28], and 1 [27] studies involved patients with CRC, rectal cancer, and colon cancer, respectively. The included studies encompassed patients from stage I to IV, with the majority involving stages II and III. Most patients underwent curative-intent surgery, and 2 [18,20] studies specifically included cases with resectable metastases. All studies conducted radiomics analysis, of which 8 [13-15,19,21,22,26,28] and 9 [10,16-18,20,23-25,27] were based on MRI and CT images, respectively. Only 1 included study applied a DL model [16], while the remaining used diverse traditional ML algorithms [10,13-15,17-28]. Regarding the number of researchers involved in image segmentation, 2 studies [24,28] had only 1 imaging researcher, 9 studies [10,13,14,17-21,23] had 2 researchers working together, 4 studies [15,22,25,26] had 3 researchers working together, and 2 studies [16,27] did not report the number of imaging researchers. Regarding the segmentation software used for ROI regions, ITK-SNAP and 3D Slicer were used in 8 [10,13-15,17,18,20,21] and 3 studies [20,24,25], respectively, with the rest of the studies using The Medical Imaging Interaction Toolkit (MITK) [16], Gold LX [22], INFINITE PACS [23], Radcloud radiomics platform, and the Eclipse system. Two studies [16,27] did not perform validation set partitioning, 1 study [22] performed 5-fold cross-validation, and 8 studies [10,13-15,17-21,25] had a separate external validation cohort [10,13-15,17-19,21], of which 5 studies [13-15,17,18] had 2 cohorts (internal and external). In 14 studies [10,13-21,23-25,28], clinical factors were combined with radiomics features to construct models, while the rest of the studies developed models using radiomics features or clinical factors alone [22,26,27]. Table S2 in Multimedia Appendix 1 [10,13-28] shows the basic characteristics of the included studies. Table S4 in Multimedia Appendix 1 [10,13-28] shows essential data for pooled analysis.

### Study Quality and RoB Assessment

The average and median RQS scores of all 17 studies were 13.23 (SD 2.56) and 13 (IQR 6-16), respectively. All studies had “Image protocol quality,” “Discrimination statistics,” and “Cut-off analyses” items. In addition, 16 [10,14-28] out of 17 studies (94%) had “Calibration statistics” and “Validation” items [10,13-26,28]. On the other hand, 13 [10,13-15,17-23,25,26] out of 17 studies (77%) conducted “Multiple segmentations.” Fourteen [10,13-21,23-25,27,28] out of 17 studies (82%) performed multivariable analysis and incorporated nonradiomics features [10,13,14,16-18,21-28], potentially yielding a more holistic model. A total of 11 [10,13,15,17-21,26-28] out of 17 studies (65%) reported potential clinical use and generated clinical decision curves. Two [15,16] studies provided open science and data access, and only 1 study [23] detected and discussed biological correlations. There were no phantom studies, and none of the studies used the comparison to the “gold standard,” adjustment for multiple testing or feature reduction, prospective designs, imaging at multiple time points, or cost-effectiveness analysis. Supplementary material provides the detailed RQS scores for all included studies (Table S3 in Multimedia Appendix 1) [10,13-28].

**Figure 2.**
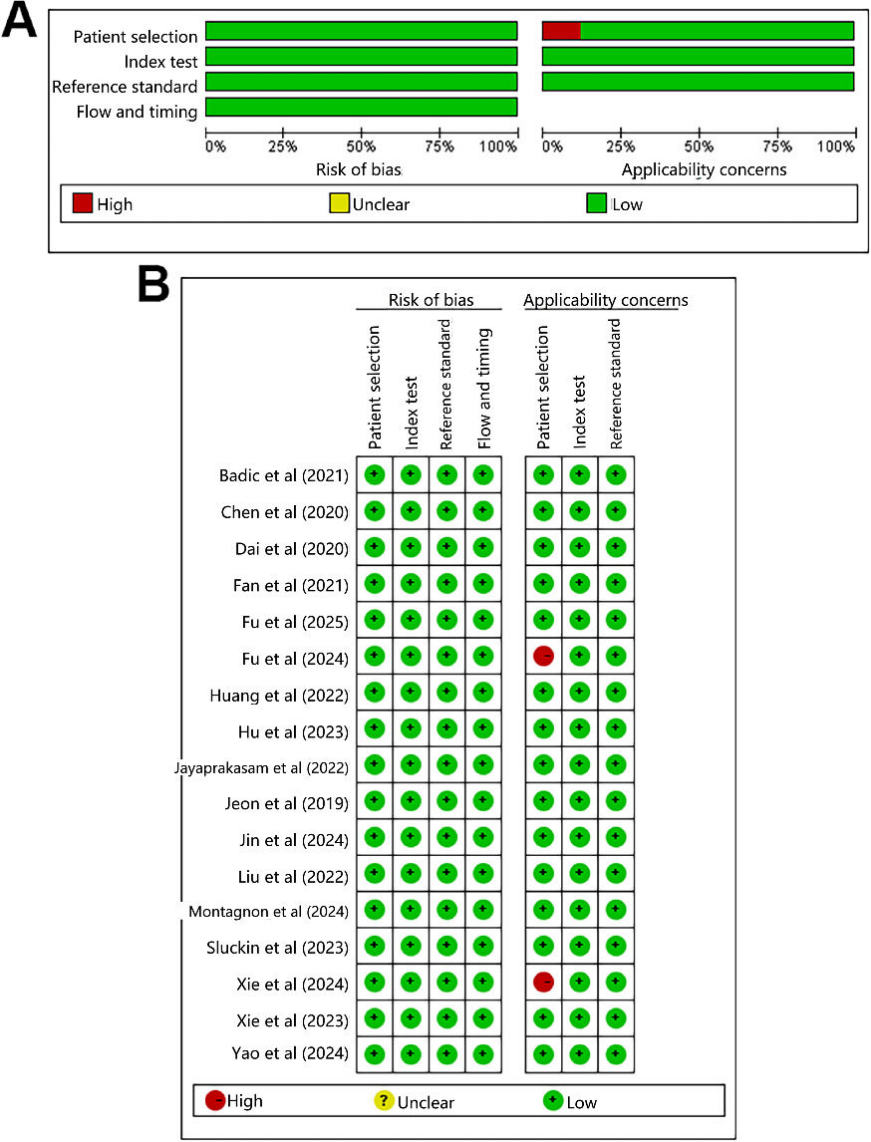
Traffic-light plot summarizing the Quality Assessment of Diagnostic Accuracy Studies-2 assessment [10,13-28].

The RoB and concerns regarding applicability for each included study were rigorously assessed using the Quality Assessment of Diagnostic Accuracy Studies (QUADAS-2) tool [33]. Notably, the included studies were cohort studies, and most of them were not excluded, which avoided the low RoB in case selection. Furthermore, considering that the included studies primarily used supervised ML with clearly defined outcomes, they had a low RoB. The implementation and interpretation of gold standards were performed by using clear criteria for recurrence, and the blinding did not influence outcomes; hence, the RoB was minimal. In addition, there was low RoB in case selection, given that known gold standards were applied. However, given that both studies enrolled cases of CRC-related metastases, there was a high RoB in the clinical applicability. A traffic-light plot summarizing the QUADAS-2 assessment for each study is presented in Figure 2.

### Meta Analysis

#### Training Set

**Figure 3.**
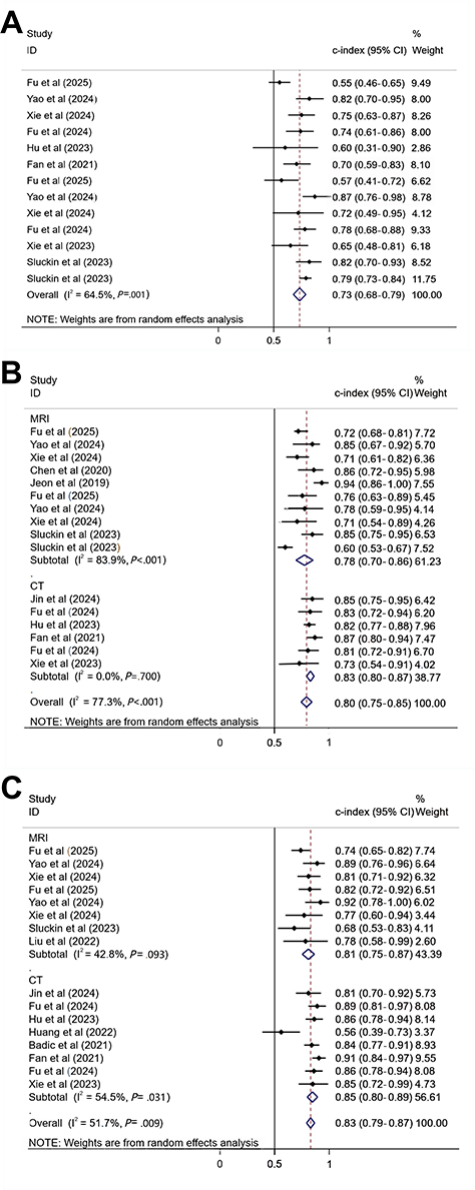
(**A**) Forest plot of c-index meta-analysis of machine learning (ML) constructed based on clinical features to predict colorectal cancer (CRC) recurrence in the training set. (**B**) Forest plot of c-index meta-analysis for ML to predict CRC recurrence based on radiomics features in the training set. (**C**) Forest plot of c-index meta-analysis of ML based on radiomics combined with clinical features to predict CRC recurrence in the training set [10,13-22,25-28]. CT: computed tomography; MRI: magnetic resonance imaging.

In total, 8 studies [10,13-15,18-20,25] compared models constructed to predict recurrence based on clinical factors, and a c-index of 0.73 (95% CI 0.69‐0.78; GRADE=low) was obtained using a random-effects model (Figure 3A; Table S5 in Multimedia Appendix 1) [10,13-28]. Furthermore, 13 radiomics-based ML models had a pooled c-index of 0.83 (95% CI 0.77‐0.89; GRADE=very low, due to inconsistency; Figure 3B; Table S5 in Multimedia Appendix 1) [10,13-28]. The c-index values of the CT-based and MRI-based radiomics summaries were 0.84 (95% CI 0.74‐0.94; GRADE=very low, due to inconsistency) and 0.81 (95% CI 0.71‐0.91; GRADE=very low, due to inconsistency), respectively. In addition, 12 studies [10,13-21,25,27] developed ML models based on radiomics features plus clinical features, with a pooled c-index of 0.82 (95% CI 0.72‐0.91; GRADE=very low, due to inconsistency; Figure 3C; Table S5 in Multimedia Appendix 1) [10,13-28].

#### Validation Set

**Figure 4.**
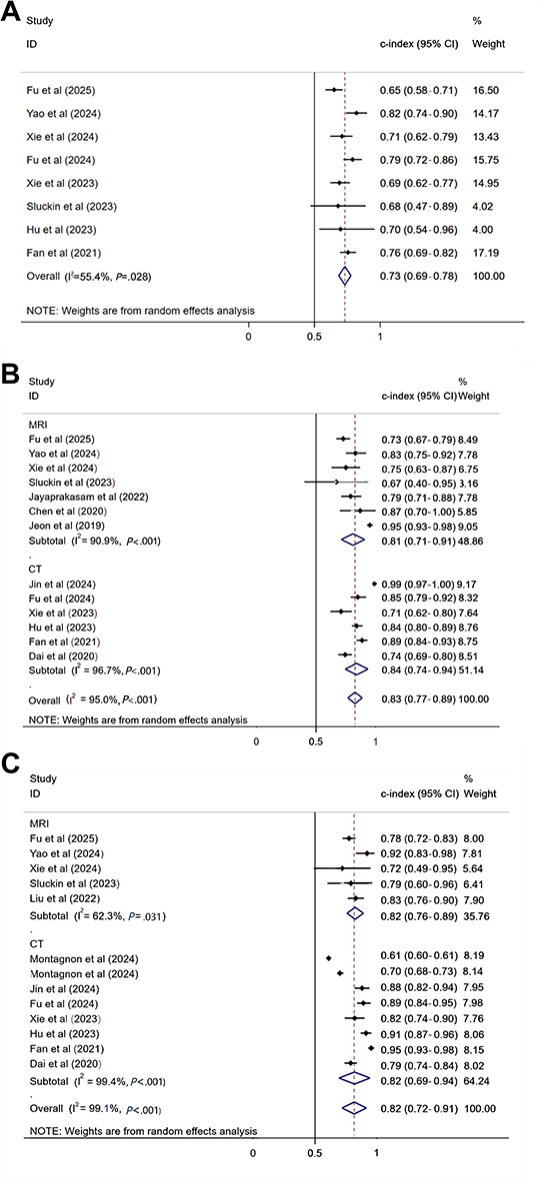
(**A**) Forest plot of c-index meta-analysis of machine learning constructed based on clinical features to predict colorectal cancer (CRC) recurrence in the validation set. (**B**) Forest plot of c-index meta-analysis for machine learning to predict CRC recurrence based on radiomics features in the validation set. (**C**) Forest plot of c-index meta-analysis of machine learning based on radiomics combined with clinical features to predict CRC recurrence in the validation set [10,13-21,23-28]. CT: computed tomography; MRI: magnetic resonance imaging.

Eight studies [10,13-15,18-20,25] compared models constructed to predict recurrence based on clinical factors, and a pooled c-index of 0.73 (95% CI 0.68‐0.79; GRADE=very low, due to inconsistency) was obtained using a random-effects model (Figure 4A; Table S5 in Multimedia Appendix 1) [10,13-28]. Publication bias was not detected by the Egger test (*P*=.11) and funnel plot (Multimedia Appendix 2). Furthermore, 16 radiomics features–based ML models had a pooled c-index of 0.80 (95% CI 0.75‐0.85; GRADE=very low, due to inconsistency; Figure 4B; Table S5 in Multimedia Appendix 1) [10,13-28]. Publication bias was not detected by Egger test (*P*=.73) and funnel plot (Multimedia Appendix 3). The c-index values of the CT-based and MRI-based radiomic summaries were 0.83 (95% CI 0.80‐0.87; GRADE=low) and 0.78 (95% CI 0.70‐0.86; GRADE=very low, due to inconsistency), respectively. In addition, ML models based on radiomics features plus clinical features had a pooled c-index of 0.83 (95% CI 0.79‐0.87; GRADE=very low, due to inconsistency; Figure 4C; Table S5 in Multimedia Appendix 1) [10,13-28]. Publication bias was detected by the Egger test (*P*=.01) and funnel plot ( Multimedia Appendix 4).

#### Internal Validation Set

Overall, 6 studies [13-15,18,20,25] compared models constructed to predict recurrence based on clinical factors, and a pooled c-index of 0.70 (95% CI 0.61‐0.79; GRADE=very low, due to inconsistency) was obtained using the random-effects model (Multimedia Appendix 5; Table S5 in Multimedia Appendix 1) [10,13-28]. Furthermore, 9 radiomics features–based ML models had a pooled c-index of 0.83 (95% CI 0.78‐0.88; GRADE=very low, due to inconsistency; Multimedia Appendix 6; Table S5 in Multimedia Appendix 1) [10,13-28]. The c-index values for the CT-based and MRI-based models were 0.84 (95% CI 0.80‐0.88; GRADE=low) and 0.82 (95% CI 0.71‐0.92; GRADE=very low, due to inconsistency), respectively. In addition, ML models based on radiomics features plus clinical features had a pooled c-index of 0.83 (95% CI 0.78‐0.88; GRADE=very low, due to inconsistency; Multimedia Appendix 7; Table S5 in Multimedia Appendix 1) [10,13-28].

#### External Validation Set

A total of 5 studies [10,14,15,18,19] compared models constructed to predict recurrence based on clinical factors and a pooled c-index of 0.76 (95% CI 0.70‐0.83; GRADE=very low, due to inconsistency) was obtained using the random-effects model (Multimedia Appendix 8; Table S5 in Multimedia Appendix 1) [10,13-28]. Furthermore, 7 ML models established using radiomics features showed a pooled c-index of 0.75 (95% CI 0.66‐0.83; GRADE=very low, due to inconsistency; Multimedia Appendix 9; Table S5 in Multimedia Appendix 1) [10,13-28], while that of MRI-based radiomics models was 0.74 (95% CI 0.62‐0.85; GRADE=very low, due to inconsistency) and that for CT-based was 0.79 (95% CI 0.71‐0.88; GRADE=low). In addition, the ML models derived from radiomics features plus clinical features had a pooled c-index of 0.83 (95% CI 0.78‐0.88; GRADE=low; Multimedia Appendix 10; Table S5 in Multimedia Appendix 1) [10,13-28].

#### Sensitivity Analysis

**Figure 5.**
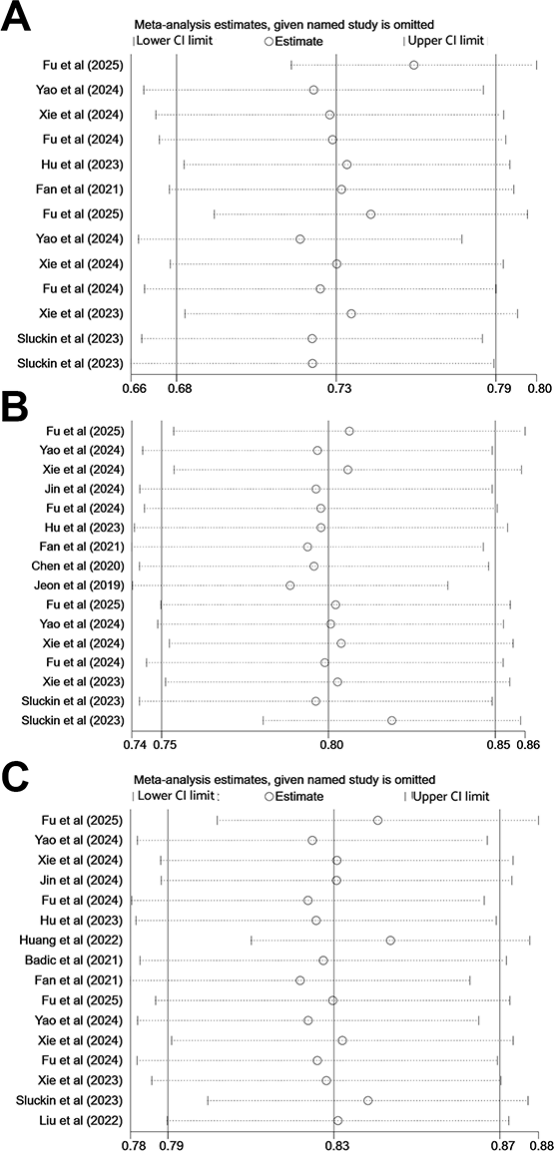
Sensitivity analysis on the validation sets based on clinical features, radiomics features, and combined clinical and radiomics features [10,13-15,17-21,23-26,28].

The robustness of the pooled estimates was determined using leave-one-out sensitivity analyses applied to the validation set results. It was observed that, while the point estimate of the pooled c-index showed minor fluctuations upon the sequential removal of each study, the overall estimates remained stable and within a consistent range. The results of the sensitivity analysis are visualized in Figure 5.

## Discussion

### Principal Findings

This meta-analysis systematically evaluated and compared the predictive performance of different radiomics-based ML models for CRC recurrence risk. We postulated that models integrating both radiomic features and clinical variables may show superior predictive accuracy compared to either modality alone. The results revealed that the integrated models achieved the highest pooled c-index of 0.83 (95% CI 0.79‐0.87) in the validation set, outperforming models based solely on radiomics features (c-index: 0.80; 95% CI 0.75‐0.85) or clinical features (c-index: 0.73; 95% CI 0.68‐0.79). Collectively, the findings of this study underscore the complementary value of combining quantitative imaging biomarkers with clinical data to enhance recurrence risk stratification in patients with CRC.

Notably, research on cancer diagnosis has seen a proliferation of systematic reviews and meta-analyses, particularly concerning CRC diagnosis, treatment response, and prognosis [34,35]. While this reflects the high clinical interest in radiomics, it also underscores the challenge of demonstrating novel and impactful contributions. Our study addresses a specific and clinically pertinent niche within this saturated field. Unlike previous reviews that often focus on a broader diagnostic or prognostic scope, this meta-analysis provides a dedicated quantitative synthesis focused exclusively on ML-based models for recurrence risk prediction. More importantly, we placed a particular emphasis on evaluating the incremental value of integrating radiomics with clinical features, a comparative approach that is less commonly the central focus of existing reviews. Therefore, we believe this work offers a nuanced and actionable insight: that the future of radiomics in clinical decision-making may lie not in replacing clinical factors, but in synergistically combining with them. This promising finding, however, should be considered in light of the detected publication bias, which indicates that the literature may lack smaller studies with negative results. Therefore, there is an urgent need for prospective validation studies in the future to confirm these promising findings.

### Comparison to Prior Work

Multiple reviews and meta-analyses have reported the clinical use of potential biomarkers in predicting CRC recurrence postsurgery [36,37]. Nonetheless, the predictive value of radiomics remains inadequately assessed. Several studies have constructed different clinical prediction models for CRC recurrence risk. For instance, Alinia constructed a predictive model and validated its predictive efficacy using 7 ML algorithms based solely on clinicopathological features (eg, disease stage and treatment type) in a cohort of 284 patients with CRC [38]. In addition, Mohammadian Rad predicted recurrence risk using a gradient-boosting model that combined clinicopathological features (eg, carcinoembryonic antigen level) and demographic data [39]. Furthermore, Seong et al [40] integrated unstructured textual information from colonoscopy reports with clinical data for CRC risk stratification.

Despite their good predictive results, these studies had some limitations, which could be attributed to several factors. First, the performance of the models based on clinical data alone in predicting CRC recurrence risk was lower than that of the fusion model integrating radiomics features, highlighting the significance of combining imaging features for an improved predictive ability. Second, colonoscopy is highly invasive and relies largely on the endoscopist’s experience and skill level; hence, its images are mainly based on morphological features, which lack quantitative analysis of deep infiltration or the tumor’s metastatic potential, thus limiting the model’s clinical applicability. Conversely, radiomics can extract hundreds of quantitative features (eg, texture and grayscale covariance matrix) from CT and MRI, among other images, reflecting tumor heterogeneity more comprehensively and providing a more reliable individualized treatment basis.

Herein, the CT and MRI images were predominantly used to predict CRC recurrence risk. Notably, there were no significant differences between the CT and MRI radiomics-based prediction models in the validation set. Nonetheless, MRI radiomics studies often require integrated multisequence image segmentation and feature extraction, whereas CT radiomics is usually based on a single modality (eg, enhanced CT) and features a relatively simplified process. In other words, MRI image segmentation has a greater workload [41]. Given that there is no significant difference in the predictive value between the 2, CT-based radiomics, despite its simplicity, can still be considered in the future in constructing radiomics-based ML prediction models.

Selecting ML algorithms remains a notable challenge when constructing radiomics-based predictive models, especially for image-processing tasks. Notably, ML models could be influenced by traditional ML and DL algorithms [42]. Traditional ML relies on the (manual) segmentation of images, image texture screening for model construction, and model validation. When extracting and screening image textures, some of the image information may be lost, somewhat impacting the model’s accuracy [43]. On the other hand, DL can intelligently segment images or be trained directly on segmented images. Furthermore, it incorporates the extraction and screening of image texture features into the training process, maximizing image information retention and providing better accuracy [44,45]. Here, manual segmentation was used owing to the few enrolled studies. Therefore, future studies should further explore the application of DL methods in constructing radiomics-based predictive models, potentially improving image recognition.

The role of clinical and demographic characteristics is particularly important when constructing prediction models based on radiomics, as they can accurately reflect disease progression. Some studies reported a significant correlation between laboratory test results and tumor staging and prognosis information, including tumor markers [46,47]. In addition, social factors such as marital status and family income were markedly linked to the prognosis of patients with tumors [48,49]. These studies highlight the potential significance of screening and incorporating meaningful clinical and sociodemographic characteristics when constructing radiomics-based prediction models. To effectively integrate these multimodal models into clinical practice, future efforts should focus on developing interoperable digital health platforms that can seamlessly combine radiomic features with real-time clinical and demographic data. Such systems should be designed to interface with existing electronic health records, support automated image analysis, and generate interpretable risk scores that can be readily used by clinicians for personalized recurrence risk assessment. In addition, implementation studies are needed to evaluate the usability, workflow integration, and clinical impact of these tools in routine care settings.

When constructing a clinical prediction model, validation is often conducted both internally and externally. For internal validation, both the training and validation sets are often derived from the same dataset and split through random sampling, among other methods [50]. Notably, internal validation often has limitations on the model’s interpretation and generalizability, especially in radiomics research that is highly dependent on images. Consequently, external validation, which involves datasets from different sources, is often recommended [51]. Herein, internal and external validation performances were assessed separately, revealing that the latter exhibited desirable prediction accuracy.

The significant statistical heterogeneity observed in our meta-analysis, while addressed by the use of a random-effects model, warrants a deeper qualitative discussion regarding its potential sources. The methodological diversity across the included studies likely introduced substantial variability that influences the interpretation of our pooled estimates. For instance, differences in imaging protocols (eg, CT vs MRI, scanner manufacturers, and contrast-enhancement phases) directly affect radiomics feature values, making it challenging to harmonize models across studies. Variations in patient characteristics, such as the inclusion of different cancer stages (I-IV), alter the underlying recurrence risk profiles of the cohorts. Simpler models, such as logistic regression, offer high interpretability but may fail to capture intricate, nonlinear relationships in the radiomics data. In contrast, more complex traditional ML algorithms (eg, random forest and support vector machines) can model these nonlinearities and often achieve higher accuracy, albeit at the cost of increased computational demand and potential overfitting if not properly regularized. While DL models hold the promise of end-to-end feature learning and potentially superior performance by automatically discovering relevant patterns from image data, they were scarcely represented in our included studies and require large datasets to train effectively. Finally, the definition of the recurrence outcome itself varied, encompassing disease-free survival, local recurrence, or time-to-recurrence, each capturing a slightly different clinical endpoint. Despite the methodological heterogeneity, our meta-analysis offers a comprehensive and robust synthesis of the current evidence. The fact that a consistently strong discriminative performance (c-index >0.80) was maintained across such varied technical and clinical contexts is a key finding, underscoring the robustness of radiomics-based prediction for CRC recurrence.

### Study Limitations

Despite its valuable insights, this study had some limitations. First, the number of eligible studies was limited, which constrained more granular subgroup analyses. In addition, the geographic origin of the evidence base was imbalanced, with 12 of the 17 included studies conducted in China. While this provides a robust assessment within that specific context, it may limit the generalizability of our findings to other populations with different genetic backgrounds, ethnicities, and healthcare systems (eg, in terms of screening protocols, treatment strategies, and staging criteria). Therefore, further multinational studies are advocated to validate the broader applicability of these radiomics models and to investigate potential geographic or ethnic variations in their performance. Second, the limited number of studies resulted in even less data for the subgroup analysis of images, possibly affecting result interpretation. Third, although subgroup analyses based on the image source (CT vs MRI) were performed in the validation set, thereby explaining some of the heterogeneity, there was significant heterogeneity. This likely reflects the clinical and methodological diversity across studies, including differences in imaging protocols, patient characteristics, model types, clinical variables, and recurrence definitions. These factors, compounded by the limited number of studies, make it difficult to quantitatively identify other potential sources of heterogeneity and highlight the challenges in achieving standardized validation across independent cohorts. Furthermore, regarding reproducibility, only 2 studies provided open-source code or datasets. This lack of transparency prevents independent validation of the proposed models and limits the clinical translation of our findings. In the future, researchers should promote open science practices by sharing code and data where possible to facilitate verification and build upon existing work. Finally, the assessment of model performance was primarily based on the c-index due to inconsistent reporting of calibration metrics (eg, Brier score and calibration slope) and time-dependent discrimination measures (eg, time-dependent–area under the curve) across studies. While the c-index provides valuable evidence of the models’ ability to stratify risk, this assessment should be complemented by future evaluations of calibration to ensure the accuracy of predicted probabilities for individual patients. Establishing robust calibration will be a crucial next step in translating these promising discriminative models into reliable clinical tools. These aspects represent important limitations that should be addressed in future studies with larger and more standardized datasets.

### Conclusions

This study demonstrates that ML models based on radiomics and incorporating clinical features exhibit superior performance in predicting the risk of CRC recurrence, with a significantly higher discriminative ability (c-index) than models relying only on a single data source. This finding highlights the significant value of multimodal data fusion in improving prediction accuracy. However, most of the existing studies use traditional ML methods that rely on manual feature extraction and screening, which may lead to information loss and limited model generalization ability. Future studies should further explore the potential of end-to-end feature learning methods, such as DL, to improve model robustness and clinical translational value by automatically extracting high-level image features and reducing manual intervention, and prioritize prospective, multicenter validation with standardized protocols and explainable AI to facilitate clinical adoption.

## Supplementary material

10.2196/78644 Multimedia Appendix 1 Data detailing the literature search strategies, characteristics of included studies, methodological quality assessments, and essential data for the meta-analysis.

10.2196/78644 Multimedia Appendix 2 Funnel plot based on clinical features in the validation set.

10.2196/78644 Multimedia Appendix 3 Funnel plot based on radiomics features in the validation set.

10.2196/78644 Multimedia Appendix 4 Funnel plot based on radiomics combined with clinical features in the validation set.

10.2196/78644 Multimedia Appendix 5 Forest plot of c-index meta-analysis of machine learning constructed based on clinical features to predict colorectal cancer recurrence in the internal validation set.

10.2196/78644 Multimedia Appendix 6 Forest plot of c-index meta-analysis for machine learning to predict colorectal cancer recurrence based on radiomics features in the internal validation set.

10.2196/78644 Multimedia Appendix 7 Forest plot of c-index meta-analysis of machine learning based on radiomics combined with clinical features to predict colorectal cancer recurrence in the internal validation set.

10.2196/78644 Multimedia Appendix 8 Forest plot of c-index meta-analysis of machine learning constructed based on clinical features to predict colorectal cancer recurrence in the external validation set.

10.2196/78644 Multimedia Appendix 9 Forest plot of c-index meta-analysis for machine learning to predict colorectal cancer recurrence based on radiomics features in the external validation set.

10.2196/78644 Multimedia Appendix 10 Forest plot of c-index meta-analysis of machine learning based on radiomics combined with clinical features to predict colorectal cancer recurrence in the external validation set.

10.2196/78644 Checklist 1 PRISMA checklist.

## Abbreviations

CRC: colorectal cancer
CT: computed tomography
DL: deep learning
GRADE: Grading of Recommendations, Assessment, Development, and Evaluation
ML: machine learning
MRI: magnetic resonance imaging
PRISMA: Preferred Reporting Items for Systematic Reviews and Meta-Analyses
PROSPERO: International Prospective Register of Systematic Reviews
RoB: risk of bias
ROI: region of interest
RQS: Radiomics Quality Score
