# Supplementary material for: Predictive Performance of Radiomics-Based Machine Learning for Colorectal Cancer Recurrence Risk: Systematic Review and Meta-Analysis
Source: JMIR Med Inform. 2025 Nov 28;13:e78644. doi: 10.2196/78644 (PMC12669921; doi:10.2196/78644)
Supplement: Multimedia Appendix 1 [file medinform-v13-e78644-s001.doc]

### Table S1. Literature search strategy

**1.Pubmed**

| Search number | Query | Results |
| --- | --- | --- |
| #1 | "Colorectal Neoplasms"[Mesh] | 251863 |
| #2 | (((((((((((((((((((((((((((((((((((Colorectal Neoplasms[Title/Abstract]) OR (Colorectal Neoplasm[Title/Abstract])) OR (Colorectal Tumors[Title/Abstract])) OR (Colorectal Tumor[Title/Abstract])) OR (Colorectal Cancer[Title/Abstract])) OR (Colorectal Cancers[Title/Abstract])) OR (Colorectal Carcinoma[Title/Abstract])) OR (Colorectal Carcinomas[Title/Abstract])) OR (colorectal tumour[Title/Abstract])) OR (colorectal tumor[Title/Abstract])) OR (Rectal Neoplasms[Title/Abstract])) OR (Rectal Neoplasm[Title/Abstract])) OR (Rectum Neoplasms[Title/Abstract])) OR (Rectum Neoplasm[Title/Abstract])) OR (Rectal Tumors[Title/Abstract])) OR (Rectal Tumor[Title/Abstract])) OR (Rectum Cancers[Title/Abstract])) OR (Rectal Cancer[Title/Abstract])) OR (Rectal Cancers[Title/Abstract])) OR (Rectum Cancer[Title/Abstract])) OR (rectum tumour[Title/Abstract])) OR (retrorectal tumor[Title/Abstract])) OR (retrorectal tumour[Title/Abstract])) OR (rectal malignancy[Title/Abstract])) OR (rectal malignancies[Title/Abstract])) OR (rectum malignancy[Title/Abstract])) OR (rectum neoplasia[Title/Abstract])) OR (Colonic Neoplasms[Title/Abstract])) OR (Colonic Neoplasm[Title/Abstract])) OR (Colon Neoplasms[Title/Abstract])) OR (Colon Neoplasm[Title/Abstract])) OR (Colon Cancers[Title/Abstract])) OR (Colonic Cancers[Title/Abstract])) OR (Colon Cancer[Title/Abstract])) OR (Colon Adenocarcinoma[Title/Abstract])) OR (Colon Adenocarcinomas[Title/Abstract]) | 248406 |
| #3 | #1 OR #2 | 329344 |
| #4 | machine learning[MeSH Terms] | 81733 |
| #5 | ((((((((((((((((((((((((((((((((machine learning[Title/Abstract]) OR (artificial intelligence[Title/Abstract])) OR (Transfer Learning[Title/Abstract])) OR (Deep learning[Title/Abstract])) OR (Ensemble Learning[Title/Abstract])) OR (prediction model[Title/Abstract])) OR (risk model[Title/Abstract])) OR (risk score[Title/Abstract])) OR (random forest[Title/Abstract])) OR (neural network[Title/Abstract])) OR (neural networks[Title/Abstract])) OR (CNN[Title/Abstract])) OR (K-Nearest Neighbor[Title/Abstract])) OR (Support vector machine[Title/Abstract])) OR (SVM[Title/Abstract])) OR (Gradient Boosting Machine[Title/Abstract])) OR (Nomogram[Title/Abstract])) OR (XGBoost[Title/Abstract])) OR (Adaboost[Title/Abstract])) OR (LightGBM[Title/Abstract])) OR (CatBoost[Title/Abstract])) OR (Gradient Boosting[Title/Abstract])) OR (Decision tree[Title/Abstract])) OR (Regression Trees[Title/Abstract])) OR (ResNet[Title/Abstract])) OR (AlexNet[Title/Abstract])) OR (VGGNet[Title/Abstract])) OR (GoogLeNet[Title/Abstract])) OR (Naive Bayesian[Title/Abstract])) OR (Multilayer perceptron[Title/Abstract])) OR (Bayesian network[Title/Abstract])) OR (Radiomics[Title/Abstract])) OR (Radiomic[Title/Abstract]) | 430880 |
| #6 | #4 OR #5 | 436653 |
| #7 | Recurrence[MeSH Terms] | 207145 |
| #8 | ((((((Recurrence[Title/Abstract]) OR (Recurrences[Title/Abstract])) OR (Recrudescence[Title/Abstract])) OR (Recrudescences[Title/Abstract])) OR (Relapse[Title/Abstract])) OR (Relapses[Title/Abstract])) OR (Recurrent[Title/Abstract])  #5 OR #6 (Recurrence[MeSH Terms]) OR (((((((Recurrence[Title/Abstract]) OR (Recurrences[Title/Abstract])) OR (Recrudescence[Title/Abstract])) OR (Recrudescences[Title/Abstract])) OR (Relapse[Title/Abstract])) OR (Relapses[Title/Abstract])) OR (Recurrent[Title/Abstract])) | 880109 |
| #9 | #7 OR #8 | 957339 |
| #10 | #3 AND #6 AND #9 | 613 |

**2.Cochrane**

| Search number | Query | Results |
| --- | --- | --- |
| #1 | MeSH descriptor: [Colorectal Neoplasms] explode all trees | 13061 |
| #2 | (Colorectal Neoplasms):ti,ab,kw OR (Colorectal Neoplasm):ti,ab,kw OR (Colorectal Tumors):ti,ab,kw OR (Colorectal Tumor):ti,ab,kw OR (Colorectal Cancer):ti,ab,kw | 21235 |
| #3 | (Colorectal Cancers):ti,ab,kw OR (Colorectal Carcinoma):ti,ab,kw OR (Colorectal Carcinomas):ti,ab,kw OR (colorectal tumour):ti,ab,kw OR (colorectal tumor):ti,ab,kw | 8466 |
| #4 | (Rectal Neoplasms):ti,ab,kw OR (Rectal Neoplasm):ti,ab,kw OR (Rectum Neoplasms):ti,ab,kw OR (Rectum Neoplasm):ti,ab,kw OR (Rectal Tumors):ti,ab,kw | 5731 |
| #5 | (Rectal Tumor):ti,ab,kw OR (Rectum Cancers):ti,ab,kw OR (Rectal Cancer):ti,ab,kw OR (Rectal Cancers):ti,ab,kw OR (Rectum Cancer):ti,ab,kw | 8818 |
| #6 | (rectum tumour):ti,ab,kw OR (retrorectal tumor):ti,ab,kw OR (retrorectal tumour):ti,ab,kw OR (rectal malignancy):ti,ab,kw OR (rectal malignancies):ti,ab,kw | 2340 |
| #7 | (rectum malignancy):ti,ab,kw OR (rectum neoplasia):ti,ab,kw OR (Colonic Neoplasms):ti,ab,kw OR (Colonic Neoplasm):ti,ab,kw OR (Colon Neoplasms):ti,ab,kw | 5369 |
| #8 | (Colon Neoplasm):ti,ab,kw OR (Colon Cancers):ti,ab,kw OR (Colonic Cancers):ti,ab,kw OR (Colon Cancer):ti,ab,kw OR (Colon Adenocarcinoma):ti,ab,kw | 7698 |
| #9 | (Colon Adenocarcinomas):ti,ab,kw | 42 |
| #10 | #1 or #2 or #3 or #4 or #5 or #6 or #7 or #8 or #9 | 31942 |
| #11 | MeSH descriptor: [Machine Learning] explode all trees | 1082 |
| #12 | (machine learning):ti,ab,kw OR (artificial intelligence):ti,ab,kw OR (Transfer Learning):ti,ab,kw OR (Deep learning):ti,ab,kw OR (Ensemble Learning):ti,ab,kw | 8180 |
| #13 | (prediction model):ti,ab,kw OR (risk model):ti,ab,kw OR (risk score):ti,ab,kw OR (random forest):ti,ab,kw OR (neural network):ti,ab,kw | 76434 |
| #14 | (neural networks):ti,ab,kw OR (CNN):ti,ab,kw OR (K-Nearest Neighbor):ti,ab,kw OR (Support vector machine):ti,ab,kw OR (SVM):ti,ab,kw | 2777 |
| #15 | (Gradient Boosting Machine):ti,ab,kw OR (Nomogram):ti,ab,kw OR (XGBoost):ti,ab,kw OR (Adaboost):ti,ab,kw OR (LightGBM):ti,ab,kw | 2157 |
| #16 | (CatBoost):ti,ab,kw OR (Gradient Boosting):ti,ab,kw OR (Decision tree):ti,ab,kw OR (Regression Trees):ti,ab,kw OR (ResNet):ti,ab,kw | 1380 |
| #17 | (AlexNet):ti,ab,kw OR (VGGNet):ti,ab,kw OR (GoogLeNet):ti,ab,kw OR (Naive Bayesian):ti,ab,kw OR (Multilayer perceptron):ti,ab,kw | 214 |
| #18 | (Bayesian network):ti,ab,kw OR (Radiomics):ti,ab,kw OR (Radiomic):ti,ab,kw | 1178 |
| #19 | #11 or #12 or #13 or #14 or #15 or #16 or #17 or #18 | 85011 |
| #20 | MeSH descriptor: [Recurrence] explode all trees | 16842 |
| #21 | (Recurrence):ti,ab,kw OR (Recurrences):ti,ab,kw OR (Recrudescence):ti,ab,kw OR (Recrudescences):ti,ab,kw OR (Relapse):ti,ab,kw | 92244 |
| #22 | (Relapses):ti,ab,kw OR (Recurrent):ti,ab,kw | 49148 |
| #23 | #20 or #21 or #22 | 119207 |
| #24 | #10 and #19 and #23 | 387 |

**3.Embase**

| Search number | Query | Results |
| --- | --- | --- |
| #1 | 'colorectal tumor'/exp | 510528 |
| #2 | 'colorectal neoplasms':ab,ti OR 'colorectal neoplasm':ab,ti OR 'colorectal tumors':ab,ti OR 'colorectal cancer':ab,ti OR 'colorectal cancers':ab,ti OR 'colorectal carcinoma':ab,ti OR 'colorectal carcinomas':ab,ti OR 'colorectal tumour':ab,ti OR 'colorectal tumor':ab,ti OR 'rectal neoplasms':ab,ti OR 'rectal neoplasm':ab,ti OR 'rectum neoplasms':ab,ti OR 'rectum neoplasm':ab,ti OR 'rectal tumors':ab,ti OR 'rectal tumor':ab,ti OR 'rectum cancers':ab,ti OR 'rectal cancer':ab,ti OR 'rectal cancers':ab,ti OR 'rectum cancer':ab,ti OR 'rectum tumour':ab,ti OR 'retrorectal tumor':ab,ti OR 'retrorectal tumour':ab,ti OR 'rectal malignancy':ab,ti OR 'rectal malignancies':ab,ti OR 'rectum malignancy':ab,ti OR 'rectum neoplasia':ab,ti OR 'colonic neoplasms':ab,ti OR 'colonic neoplasm':ab,ti OR 'colon neoplasms':ab,ti OR 'colon neoplasm':ab,ti OR 'colon cancers':ab,ti OR 'colonic cancers':ab,ti OR 'colon cancer':ab,ti OR 'colon adenocarcinoma':ab,ti OR 'colon adenocarcinomas':ab,ti | 350504 |
| #3 | 'machine learning'/exp | 536882 |
| #4 | 'machine learning':ab,ti OR 'artificial intelligence':ab,ti OR 'transfer learning':ab,ti OR 'deep learning':ab,ti OR 'ensemble learning':ab,ti OR 'prediction model':ab,ti OR 'risk model':ab,ti OR 'risk score':ab,ti OR 'colorectal tumour':ab,ti OR 'random forest':ab,ti OR 'neural network':ab,ti OR 'neural networks':ab,ti OR cnn:ab,ti OR 'k-nearest neighbor':ab,ti OR 'support vector machine':ab,ti OR svm:ab,ti OR 'gradient boosting machine':ab,ti OR nomogram:ab,ti OR xgboost:ab,ti OR adaboost:ab,ti OR lightgbm:ab,ti OR catboost:ab,ti OR 'gradient boosting':ab,ti OR 'decision tree':ab,ti OR 'regression trees':ab,ti OR resnet:ab,ti OR alexnet:ab,ti OR vggnet:ab,ti OR googlenet:ab,ti OR 'naive bayesian':ab,ti OR 'multilayer perceptron':ab,ti OR 'bayesian network':ab,ti OR radiomics:ab,ti OR radiomic:ab,ti | 567552 |
| #5 | recurrence:ab,ti OR recurrences:ab,ti OR recrudescence:ab,ti OR recrudescences:ab,ti OR relapse:ab,ti OR relapses:ab,ti OR recurrent:ab,ti | 1355840 |
| #6 | #1 OR #2 | 543428 |
| #7 | #3 OR #4 | 821400 |
| #8 | #5 AND #6 AND #7 | 1282 |

**4.Web of Science**

| Search number | Query | Results |
| --- | --- | --- |
| #1 | Colorectal Neoplasms (Topic) OR Colorectal Neoplasm (Topic) OR Colorectal Tumors (Topic) OR Colorectal Tumor (Topic) OR Colorectal Cancer (Topic) OR Colorectal Cancers (Topic) OR Colorectal Carcinoma (Topic) OR Colorectal Carcinomas (Topic) OR colorectal tumour (Topic) OR colorectal tumor (Topic) OR Rectal Neoplasms (Topic) OR Rectal Neoplasm (Topic) OR Rectum Neoplasms (Topic) OR Rectum Neoplasm (Topic) OR Rectal Tumors (Topic) OR Rectal Tumor (Topic) OR Rectum Cancers (Topic) OR Rectal Cancer (Topic) OR Rectal Cancers (Topic) OR Rectum Cancer (Topic) OR rectum tumour (Topic) OR retrorectal tumor (Topic) OR retrorectal tumour (Topic) OR rectal malignancy (Topic) OR rectal malignancies (Topic) OR rectum malignancy (Topic) OR rectum neoplasia (Topic) OR Colonic Neoplasms (Topic) OR Colonic Neoplasm (Topic) OR Colon Neoplasms (Topic) OR Colon Neoplasm (Topic) OR Colon Cancers (Topic) OR Colonic Cancers (Topic) OR Colon Cancer (Topic) OR Colon Adenocarcinoma (Topic) OR Colon Adenocarcinomas (Topic) | 338626 |
| #2 | machine learning (Topic) OR artificial intelligence (Topic) OR Transfer Learning (Topic) OR Deep learning (Topic) OR Ensemble Learning (Topic) OR prediction model (Topic) OR risk model (Topic) OR risk score (Topic) OR random forest (Topic) OR neural network (Topic) OR neural networks (Topic) OR CNN (Topic) OR K-Nearest Neighbor (Topic) OR Support vector machine (Topic) OR SVM (Topic) OR Gradient Boosting Machine (Topic) OR Nomogram (Topic) OR XGBoost (Topic) OR Adaboost (Topic) OR LightGBM (Topic) OR CatBoost (Topic) OR Gradient Boosting (Topic) OR Decision tree (Topic) OR Regression Trees (Topic) OR ResNet (Topic) OR AlexNet (Topic) OR VGGNet (Topic) OR GoogLeNet (Topic) OR Naive Bayesian (Topic) OR Multilayer perceptron (Topic) OR Bayesian network (Topic) OR Radiomics (Topic) OR Radiomic (Topic) | 2650142 |
| #3 | Recurrence (Topic) OR Recurrences (Topic) OR Recrudescence (Topic) OR Recrudescences (Topic) OR Relapse (Topic) OR Relapses (Topic) OR Recurrent (Topic) | 768874 |
| #4 | #3 AND #2 AND #1 | 3634 |

### Table S2. Basic characteristics of the included studies.

| **No.** | **First author** | **Years of publication** | **Country** | **Source of patients** | **Type of patients** | **Treatment programme** | **Radiomics source** | **Number of imaging researchers** | **ROI extraction software** | **Total number of cases** | **Number of cases in the training set** | **Outcome indicators and clinical definition** | **Validation set generation method** | **Number of cases in the validation set** | **Type of model used** | **Modelling variables** |
| --- | --- | --- | --- | --- | --- | --- | --- | --- | --- | --- | --- | --- | --- | --- | --- | --- |
| 1 | Fu et al [13] | 2025 | China | Multicenter | pT stage 0-4 rectal cancer | Curative resection | T2/DWI MRI | 2 | ITK-SNAP | 600 | 358 | ^c^DFS | IVS^a^:random sampling; EVS^b^:independent institutions | IVS:120 EVS:122 | Cox regression | Clinical features;Radiomics features;Radiomics combined with clinical features |
| 2 | Yao et al [14] | 2024 | China | Multicenter | pT stage 1-4 rectal adenocarcinoma | Curative resection | CE-T1/T2/DWI MRI | 2 | ITK-SNAP | 234 | 129 | DFS | IVS:random sampling; EVS:independent institutions | IVS:55 EVS:50 | Logistic regression | Clinical features;Radiomics features;Radiomics combined with clinical features |
| 3 | Xie et al [15] | 2024 | China | Multicenter | pTNM stage 0-III rectal cancer | Radical surgery | T2/DWI MRI | 3 | ITK-SNAP | 264 | 158 | DFS | IVS:random sampling; EVS:independent institutions | IVS:65 EVS:41 | Cox regression | Clinical features;Radiomics features;Radiomics combined with clinical features |
| 4 | Montagnon et al [16] | 2024 | Canada | Single-center | Colorectal cancer | Systemic chemotherapy and surgery | Portal venous CT | - | MITK | 205 | 205 | TTR^d^ | ~~-~~ | ~~-~~ | Random Survival Forest;DeepSurv | Radiomics combined with clinical features |
| 5 | Jin et al [17] | 2024 | China | Multicenter | T stage 1-4 colorectal cancer | Curative resection | Portal venous CT | 2 | ITK-SNAP | 220 | 108 | 3-year early recurrence | IVS:random sampling; EVS:independent institutions | IVS:45 EVS:67 | Logistic Regression**;**Adaptive Boosting;Multi-Layer Perceptron;Naive Bayes;Random Forest | Radiomics combined with clinical features |
| 6 | Fu et al [18] | 2024 | China | Multicenter | T stage 1-4 colorectal cancer with liver metastases | Curative resection | Portal venous/delayed phase CT | 2 | ITK-SNAP | 282 | 138 | 3-year DFS | IVS:random sampling; EVS:independent institutions | IVS:60 EVS:84 | Logistic regression | Clinical features;Radiomics features;Radiomics combined with clinical features |
| 7 | Xie et al [10] | 2023 | China | Multicenter | TNM stage I-IV rectal cancer | Curative resection | Portal venous CT | 2 | ITK-SNAP | 405 | 298 | 3-year DFS | EVS:independent institutions | EVS:107 | Logistic regression | Clinical features;Radiomics features;Radiomics combined with clinical features |
| 8 | Sluckin et al [19] | 2023 | Netherlands | Multicenter | T stage 2-4 rectal cancer | Total mesorectal excision | T2WI MRI | 2 | 3D Slicer | 196 | 172 | LR | EVS:independent institutions | EVS:24 | AdaBoost | Clinical features;Radiomics features;Radiomics combined with clinical features |
| 9 | Hu et al [20] | 2023 | China | Single-center | TNM stage I-IV colorectal cancer with liver metastases | Microwave ablation | Portal venous CT | 2 | ITK-SNAP | 318 | 216 | local tumor progression | IVS:random sampling | IVS:102 | support vector machine | Clinical features;Radiomics features;Radiomics combined with clinical features |
| 10 | Liu et al [21] | 2022 | China | Multicenter | pT stage 1-4 rectal cancer | Total mesorectal excision | T2/DWI MRI | 2 | ITK-SNAP | 195 | 158 | DFS | EVS:independent institutions | EVS:37 | support vector machine | Radiomics combined with clinical features |
| 11 | Jayaprakasam et al [22] | 2022 | USA | Single-center | cTNM stage 2-3 rectal cancer | Total mesorectal excision | T2WI MRI | 3 | Gold LX | 236 | - | LR^e^/DR^f^ | 5-fold cross-validation | - | support vector machine | Radiomics features |
| 12 | Huang et al [23] | 2022 | Taiwan | Single-center | Stage III colorectal cancer | Surgery and adjuvant chemotherapy | Abdominal/pelvic CT | 2 | INFINITE PACS | 71 | 47 | DFS | IVS:random sampling | IVS:24 | Random Forest | Radiomics combined with clinical features |
| 13 | Badic et al [24] | 2021 | France | Multicenter | Stage II and III colorectal cancer | Surgery and adjuvant chemotherapy | Portal venous CT | 1 | 3D Slicer | 193 | 136 | DFS | IVS:random sampling | IVS:57 | Logistic Regression;support vector machine;Random Forest | Radiomics combined with clinical features |
| 14 | Fan et al [25] | 2021 | China | Single-center | Stage II Colorectal cancer | Surgical  resection | Portal venous CT | 3 | 3D Slicer | 299 | 210 | 3-year DFS | IVS:random sampling | IVS:89 | Cox | Clinical features;Radiomics features;Radiomics combined with clinical features |
| 15 | Chen et al [26] | 2020 | China | Single-center | pTNM stage 0-III rectal cancer | Total mesorectal excision | T2/DWI MRI | 3 | Radcloud radiomics platform | 80 | 40 | LR | IVS:random sampling | IVS:40 | XGBoost | Radiomics features |
| 16 | Dai et al [27] | 2020 | China | Single-center | Stage I-III Colon cancer | Radical primary resection | Contrast-enhanced abdominal pelvic CT | - | - | 701 | 701 | DFS | - | - | Cox regression | Radiomics features |
| 17 | Jeon et al [28] | 2019 | Republic of Korea | Single-center | pT stage 0-4 rectal cancer | Preoperative chemoradiotherapy and total mesorectal excision | T2/DWI MRI | 1 | Eclipse system | 101 | 67 | DFS | IVS:random sampling | IVS:34 | Cox regression | Radiomics combined with clinical features |

^a^IVS: Internal validation set; ^b^EVS: External validation set; ^c^DFS: disease-free survival, as the time between radical resection and local recurrence or distant metastasis; ^d^TTR: time to recurrence; ^e^LR: local recurrence; ^f^DR: distant recurrence.

### Table S3. Methodology quality assessment of each study by the RQS tool

| **Study No.** | **Image protocol quality** | **Multiple segmentations** | **Phantom study on all scanners** | **Imaging at multiple time points** | **Feature reduction** | **Multivariable analysis** | **Biological correlation** | **Cut-off analyses** | **Discrimination statistics** | **Calibration statistics** | **Prospective study** | **Validation** | **Comparison to 'gold standard'** | **Potential clinical utility** | **Cost-effectiveness analysis** | **Open science and data** | **Total points** |
| --- | --- | --- | --- | --- | --- | --- | --- | --- | --- | --- | --- | --- | --- | --- | --- | --- | --- |
| Fu et al [13] 2025 | 1 | 1 | 0 | 0 | 3 | 1 | 0 | 1 | 1 | 0 | 0 | 3 | 0 | 2 | 0 | 0 | 13(36.11%) |
| Yao et al [14] 2024 | 1 | 1 | 0 | 0 | 3 | 1 | 0 | 1 | 2 | 2 | 0 | 3 | 0 | 0 | 0 | 0 | 14(38.89%) |
| Xie et al [15] 2024 | 1 | 1 | 0 | 0 | 3 | 1 | 0 | 1 | 1 | 1 | 0 | 3 | 0 | 2 | 0 | 1 | 15(41.67%) |
| Montagnon et al [16] 2024 | 1 | 0 | 0 | 0 | 3 | 1 | 0 | 1 | 2 | 1 | 0 | 2 | 0 | 0 | 0 | 1 | 12(33.33%) |
| Jin et al [17] 2024 | 1 | 1 | 0 | 0 | 3 | 1 | 0 | 1 | 2 | 2 | 0 | 3 | 0 | 2 | 0 | 0 | 16(44.44%) |
| Fu et al [18] 2024 | 1 | 1 | 0 | 0 | 3 | 1 | 0 | 1 | 1 | 1 | 0 | 3 | 0 | 2 | 0 | 0 | 14(38.89%) |
| Xie et al [10] 2023 | 1 | 1 | 0 | 0 | 3 | 1 | 0 | 1 | 2 | 2 | 0 | 3 | 0 | 2 | 0 | 0 | 16(44.44%) |
| Sluckin et al [19] 2023 | 1 | 1 | 0 | 0 | 3 | 1 | 0 | 1 | 2 | 2 | 0 | 3 | 0 | 2 | 0 | 0 | 16(44.44%) |
| Hu et al [20] 2023 | 1 | 1 | 0 | 0 | 3 | 1 | 0 | 1 | 2 | 2 | 0 | 2 | 0 | 2 | 0 | 0 | 15(41.67%) |
| Liu et al [21] 2022 | 1 | 1 | 0 | 0 | 3 | 1 | 0 | 1 | 2 | 2 | 0 | 3 | 0 | 2 | 0 | 0 | 16(44.44%) |
| Jayaprakasam et al [22] 2022 | 1 | 1 | 0 | 0 | 3 | 0 | 0 | 1 | 2 | 1 | 0 | 2 | 0 | 0 | 0 | 0 | 11(30.56%) |
| Huang et al [23] 2022 | 1 | 1 | 0 | 0 | 3 | 1 | 1 | 1 | 2 | 1 | 0 | 2 | 0 | 0 | 0 | 0 | 13(36.11%) |
| Badic et al [24] 2021 | 1 | 0 | 0 | 0 | 3 | 1 | 0 | 1 | 2 | 1 | 0 | 2 | 0 | 0 | 0 | 0 | 11(30.56%) |
| Fan et al [25] 2021 | 1 | 1 | 0 | 0 | 3 | 1 | 0 | 1 | 2 | 1 | 0 | 2 | 0 | 0 | 0 | 0 | 12(33.33%) |
| Chen et al [26] 2020 | 1 | 1 | 0 | 0 | 3 | 0 | 0 | 1 | 2 | 1 | 0 | 2 | 0 | 2 | 0 | 0 | 13(36.11%) |
| Dai et al [27] 2020 | 1 | 0 | 0 | 0 | 3 | 1 | 0 | 1 | 2 | 1 | 0 | -5 | 0 | 2 | 0 | 0 | 6(16.67%) |
| Jeon et al [28] 2019 | 1 | 0 | 0 | 0 | 3 | 0 | 0 | 1 | 2 | 1 | 0 | 2 | 0 | 2 | 0 | 0 | 12(33.33%) |

### Table S4. Essential data for pooled analysis.

| Study No. | Image | Dataset | No. of events | Sample size | Variable | c-index | SE |
| --- | --- | --- | --- | --- | --- | --- | --- |
| Fu et al [13] 2025 | MRI | Training set | 63 | 358 | Clinical features | 0.6500 | 0.037 |
| Fu et al [13] 2025 | MRI | Training set | 63 | 358 | Radiomics + Clinical features | 0.7750 | 0.027 |
| Fu et al [13] 2025 | MRI | Training set | 63 | 358 | Radiomics features | 0.7280 | 0.030 |
| Fu et al [13] 2025 | MRI | Internal validation set | 26 | 120 | Radiomics features | 0.7200 | 0.055 |
| Fu et al [13] 2025 | MRI | Internal validation set | 26 | 120 | Radiomics features+Clinical features | 0.7390 | 0.054 |
| Fu et al [13] 2025 | MRI | Internal validation set | 26 | 120 | Clinical features | 0.5520 | 0.062 |
| Fu et al [13] 2025 | MRI | External validation set | 11 | 122 | Radiomics features | 0.7580 | 0.068 |
| Fu et al [13] 2025 | MRI | External validation set | 11 | 122 | Radiomics features+Clinical features | 0.8220 | 0.052 |
| Fu et al [13] 2025 | MRI | External validation set | 11 | 122 | Clinical features | 0.5690 | 0.079 |
| Yao et al [14] 2024 | MRI | Training set | 23 | 129 | Clinical features | 0.8200 | 0.048 |
| Yao et al [14] 2024 | MRI | Training set | 23 | 129 | Radiomics + Clinical features | 0.9200 | 0.046 |
| Yao et al [14] 2024 | MRI | Training set | 23 | 129 | Radiomics features | 0.8300 | 0.041 |
| Yao et al [14] 2024 | MRI | Internal validation set | 14 | 55 | Radiomics features | 0.8500 | 0.059 |
| Yao et al [14] 2024 | MRI | Internal validation set | 14 | 55 | Clinical features | 0.8200 | 0.064 |
| Yao et al [14] 2024 | MRI | Internal validation set | 14 | 55 | Radiomics features+Clinical features | 0.8900 | 0.051 |
| Yao et al [14] 2024 | MRI | External validation set | 12 | 50 | Radiomics features | 0.7800 | 0.097 |
| Yao et al [14] 2024 | MRI | External validation set | 12 | 50 | Clinical features | 0.8700 | 0.056 |
| Yao et al [14] 2024 | MRI | External validation set | 12 | 50 | Radiomics features+Clinical features | 0.9200 | 0.071 |
| Xie et al [15] 2024 | MRI | Training set | - | 158 | Clinical features | 0.7100 | 0.046 |
| Xie et al [15] 2024 | MRI | Training set | - | 158 | Radiomics + Clinical features | 0.7200 | 0.117 |
| Xie et al [15] 2024 | MRI | Training set | - | 158 | Radiomics features | 0.7500 | 0.061 |
| Xie et al [15] 2024 | MRI | Internal validation set | - | 65 | Radiomics features | 0.7100 | 0.051 |
| Xie et al [15] 2024 | MRI | internal validation set | - | 65 | Clinical features | 0.750 | 0.061 |
| Xie et al [15] 2024 | MRI | internal validation set | - | 65 | Radiomics features+Clinical features | 0.8100 | 0.051 |
| Xie et al [15] 2024 | MRI | External validation set | - | 41 | Radiomics features | 0.7100 | 0.087 |
| Xie et al [15] 2024 | MRI | External validation set | - | 41 | Clinical features | 0.7200 | 0.117 |
| Xie et al [15] 2024 | MRI | External validation set | - | 41 | Radiomics features+Clinical features | 0.7700 | 0.087 |
| Montagnon et al [16] 2024 | CT | Training set | 41 | 205 | Radiomics + Clinical features | 0.6100 | 0.005 |
| Montagnon et al [16] 2024 | CT | Training set | 41 | 205 | Radiomics + Clinical features | 0.7000 | 0.010 |
| Jin et al [17] 2024 | CT | Training set | 48 | 108 | Radiomics + Clinical features | 0.8800 | 0.030 |
| Jin et al [17] 2024 | CT | Training set | 48 | 108 | Radiomics features | 0.9900 | 0.009 |
| Jin et al [17] 2024 | CT | Internal validation set | 20 | 45 | Radiomics features | 0.8500 | 0.053 |
| Jin et al [17] 2024 | CT | Internal validation set | 20 | 45 | Radiomics features+Clinical features | 0.8100 | 0.059 |
| Fu et al [13] 2024 | CT | Training set | - | 138 | Clinical features | 0.7900 | 0.036 |
| Fu et al [13] 2024 | CT | Training set | - | 138 | Radiomics + Clinical features | 0.8900 | 0.026 |
| Fu et al [13] 2024 | CT | Training set | - | 138 | Radiomics features | 0.8500 | 0.031 |
| Fu et al [13] 2024 | CT | Internal validation set | - | 60 | Radiomics features | 0.8300 | 0.056 |
| Fu et al [13] 2024 | CT | Internal validation set | - | 60 | Clinical features | 0.7400 | 0.066 |
| Fu et al [13] 2024 | CT | Internal validation set | - | 60 | Radiomics features+Clinical features | 0.8900 | 0.041 |
| Fu et al [13] 2024 | CT | External validation set | - | 84 | Radiomics features | 0.8100 | 0.046 |
| Fu et al [13] 2024 | CT | External validation set | - | 84 | Clinical features | 0.7800 | 0.051 |
| Fu et al [13] 2024 | CT | External validation set | - | 84 | Radiomics features+Clinical features | 0.8600 | 0.041 |
| Xie et al [10] 2023 | CT | Training set | 41 | 298 | Clinical features | 0.6900 | 0.036 |
| Xie et al [10] 2023 | CT | Training set | 41 | 298 | Radiomics + Clinical features | 0.8200 | 0.041 |
| Xie et al [10] 2023 | CT | Training set | 41 | 298 | Radiomics features | 0.7100 | 0.046 |
| Xie et al [10] 2023 | CT | External validation set | 10 | 107 | Radiomics features | 0.7300 | 0.097 |
| Xie et al [10] 2023 | CT | External validation set | 10 | 107 | Clinical features | 0.6500 | 0.087 |
| Xie et al [10] 2023 | CT | External validation set | 10 | 107 | Radiomics features+Clinical features | 0.8500 | 0.066 |
| Sluckin et al [19] 2023 | MRI | Training set | - | 172 | Clinical features | 0.6800 | 0.107 |
| Sluckin et al [19] 2023 | MRI | Training set | - | 172 | Radiomics + Clinical features | 0.7900 | 0.097 |
| Sluckin et al [19] 2023 | MRI | Training set | - | 172 | Radiomics features | 0.6700 | 0.138 |
| Sluckin et al [19] 2023 | MRI | External validation set | - | 24 | Radiomics features | 0.8500 | 0.051 |
| Sluckin et al [19] 2023 | MRI | External validation set | - | 24 | Clinical features | 0.8200 | 0.061 |
| Sluckin et al [19] 2023 | MRI | External validation set | - | 24 | Radiomics features+Clinical features | 0.6800 | 0.077 |
| Sluckin et al [19] 2023 | MRI | External validation set | - | 24 | Radiomics features | 0.6000 | 0.036 |
| Sluckin et al [19] 2023 | MRI | External validation set | - | 24 | Clinical features | 0.7900 | 0.031 |
| Hu et al [20] 2023 | CT | Training set | 58 | 216 | Clinical features | 0.6980 | 0.083 |
| Hu et al [20] 2023 | CT | Training set | 58 | 216 | Radiomics + Clinical features | 0.9120 | 0.022 |
| Hu et al [20] 2023 | CT | Training set | 58 | 216 | Radiomics features | 0.8400 | 0.020 |
| Hu et al [20] 2023 | CT | Internal validation set | 27 | 102 | Radiomics features | 0.8200 | 0.026 |
| Hu et al [20] 2023 | CT | Internal validation set | 27 | 102 | Clinical features | 0.6020 | 0.148 |
| Hu et al [20] 2023 | CT | Internal validation set | 27 | 102 | Radiomics features+Clinical features | 0.8630 | 0.040 |
| Liu et al [21] 2022 | MRI | Training set | 31 | 158 | Radiomics + Clinical features | 0.8290 | 0.033 |
| Liu et al [21] 2022 | MRI | External validation set | 9 | 37 | Radiomics features+Clinical features | 0.7830 | 0.104 |
| Jayaprakasam et al [22] 2022 | MRI | Training set | 42 | 236 | Radiomics features | 0.7900 | 0.041 |
| Huang et al [23] 2022 | CT | Internal validation set | 7 | 24 | Radiomics features+Clinical features | 0.5600 | 0.088 |
| Badic et al [24] 2022 | CT | internal validation set | 14 | 57 | Radiomics features+Clinical features | 0.8400 | 0.035 |
| Fan et al [25] 2021 | CT | Training set | - | 210 | Clinical features | 0.7560 | 0.032 |
| Fan et al [25] 2021 | CT | Training set | - | 210 | Radiomics + Clinical features | 0.9540 | 0.012 |
| Fan et al [25] 2021 | CT | Training set | - | 210 | Radiomics features | 0.8860 | 0.023 |
| Fan et al [25] 2021 | CT | Internal validation set | - | 89 | Radiomics features | 0.8740 | 0.037 |
| Fan et al [25] 2021 | CT | Internal validation set | - | 89 | Clinical features | 0.7050 | 0.061 |
| Fan et al [25] 2021 | CT | Internal validation set | - | 89 | Radiomics features+Clinical features | 0.9060 | 0.032 |
| Chen et al [26] 2020 | MRI | Training set | 5 | 40 | Radiomics features | 0.8700 | 0.087 |
| Chen et al [26] 2020 | MRI | Internal validation set | 6 | 40 | Radiomics features | 0.8640 | 0.074 |
| Dai et al [27] 2020 | CT | Training set | 351 | 701 | Radiomics + Clinical features | 0.7880 | 0.026 |
| Dai et al [27] 2020 | CT | Training set | 351 | 701 | Radiomics features | 0.7440 | 0.029 |
| Jeon et al [28] 2019 | MRI | Training set | - | 67 | Radiomics features | 0.9510 | 0.013 |
| Jeon et al [28] 2019 | MRI | Internal validation set | - | 34 | Radiomics features | 0.9370 | 0.038 |

### Table S5. Prediction Interval and GRADE Rating in the training set and the validation set

| Subgroup Analysis | Training set | | | | |  | Validation set | | | | |
| --- | --- | --- | --- | --- | --- | --- | --- | --- | --- | --- | --- |
|  | n | c-index (95%CI) | 95% prediction interval | I^2^ | Grade |  | n | c-index (95%CI) | 95% prediction interval | I^2^ | Grade |
| Clinical features | 8 | 0.73(0.69-0.78) | 0.61-0.87 | 55.40 | ⊕⊕ΘΘ |  | 13 | 0.73(0.68-0.79) | 0.58-0.94 | 64.50 | ⊕ΘΘΘ |
| Radiomics features |  |  |  |  |  |  |  |  |  |  |  |
| CT | 6 | 0.84(0.74-0.94) | 0.61-1.00 | 96.70 | ⊕ΘΘΘ |  | 6 | 0.83(0.80-0.87) | 0.79-0.88 | 0 | ⊕⊕ΘΘ |
| MRI | 7 | 0.81(0.71-0.91) | 0.63-1.00 | 90.90 | ⊕ΘΘΘ |  | 10 | 0.78(0.70-0.86) | 0.58-1.00 | 83.90 | ⊕ΘΘΘ |
| Overall | 13 | 0.83(0.77-0.89) | 0.66-1.00 | 95.00 | ⊕ΘΘΘ |  | 16 | 0.80(0.75-0.85) | 0.64-0.99 | 76.40 | ⊕ΘΘΘ |
| Clinical and radiomics features |  |  |  |  |  |  |  |  |  |  |  |
| CT | 8 | 0.82(0.69-0.94) | 0.55-1.000 | 99.40 | ⊕ΘΘΘ |  | 8 | 0.85(0.80-0.89) | 0.83-0.90 | 54.50 | ⊕ΘΘΘ |
| MRI | 5 | 0.82(0.76-0.89) | 0.66-1.000 | 62.30 | ⊕ΘΘΘ |  | 8 | 0.81(0.75-0.87) | 0.69-0.96 | 42.80 | ⊕⊕ΘΘ |
| Overall | 13 | 0.82(0.72-0.91) | 0.61-1.00 | 99.10 | ⊕ΘΘΘ |  | 16 | 0.83(0.79-0.87) | 0.77-0.92 | 51.70 | ⊕ΘΘΘ |

| Subgroup Analysis | Internal validation set | | | | |  | External validation set | | | | |
| --- | --- | --- | --- | --- | --- | --- | --- | --- | --- | --- | --- |
|  | n | c-index (95%CI) | 95% prediction interval | I^2^ | Grade |  | n | c-index (95%CI) | 95% prediction interval | I^2^ | Grade |
| Clinical features | 6 | 0.70(0.61-0.79) | 0.50-0.99 | 64.00 | ⊕ΘΘΘ |  | 7 | 0.76(0.70-0.83) | 0.74-0.84 | 53.70 | ⊕ΘΘΘ |
| Radiomics features |  |  |  |  |  |  |  |  |  |  |  |
| CT | 4 | 0.84(0.80-0.88) | 0.78-0.91 | 0 | ⊕⊕ΘΘ |  | 2 | 0.79(0.71-0.88) | 0.40-1.00 | 0 | ⊕⊕ΘΘ |
| MRI | 5 | 0.82(0.71-0.92) | 0.57-1.00 | 84.30 | ⊕ΘΘΘ |  | 5 | 0.74(0.62-0.85) | 0.49-1.00 | 77.80 | ⊕ΘΘΘ |
| Overall | 9 | 0.83(0.78-0.88) | 0.69-1.00 | 71.30 | ⊕ΘΘΘ |  | 7 | 0.75(0.66-0.83) | 0.55-1.00 | 72.70 | ⊕ΘΘΘ |
| Clinical and radiomics features |  |  |  |  |  |  |  |  |  |  |  |
| CT | 6 | 0.84(0.78-0.90) | 0.81-0.93 | 67.50 | ⊕ΘΘΘ |  | 2 | 0.86(0.79-0.93) | 0.51-1.00 | 0 | ⊕⊕ΘΘ |
| MRI | 3 | 0.81(0.72-0.90) | 0.55-1.00 | 61.00 | ⊕ΘΘΘ |  | 5 | 0.81(0.73-0.89) | 0.63-1.00 | 42.70 | ⊕⊕ΘΘ |
| Overall | 9 | 0.83(0.78-0.88) | 0.73-0.96 | 65.60 | ⊕ΘΘΘ |  | 7 | 0.83(0.78-0.88) | 0.79-0.90 | 22.00 | ⊕⊕ΘΘ |

### References

10. Xie Z, Zhang Q, Wang X, Chen Y, Deng Y, Lin H, et al. Development and validation of a novel radiomics nomogram for prediction of early recurrence in colorectal cancer. Eur J Surg Oncol. 2023 Dec;49(12):107118. PMID: 37844471. doi: 10.1016/j.ejso.2023.107118.

13. Fu S, Xia T, Li Z, Zhu J, Zeng Z, Li B, et al. Baseline MRI-based radiomics improving the recurrence risk stratification in rectal cancer patients with negative carcinoembryonic antigen: A multicenter cohort study. Eur J Radiol. 2025 Jan;182:111839. PMID: 39591940. doi: 10.1016/j.ejrad.2024.111839.7

14. Yao X, Zhu X, Deng S, Zhu S, Mao G, Hu J, et al. MRI-based radiomics for preoperative prediction of recurrence and metastasis in rectal cancer. Abdom Radiol (NY). 2024 Apr;49(4):1306-19. PMID: 38407804. doi: 10.1007/s00261-024-04205-y.

15. Xie PY, Zeng ZM, Li ZH, Niu KX, Xia T, Ma DC, et al. MRI-based radiomics for stratifying recurrence risk of early-onset rectal cancer: a multicenter study. ESMO Open. 2024 Oct;9(10):103735. PMID: 39368416. doi: 10.1016/j.esmoop.2024.103735.

16. Montagnon E, Cerny M, Hamilton V, Derennes T, Ilinca A, Elforaici MEA, et al. Radiomics analysis of baseline computed tomography to predict oncological outcomes in patients treated for resectable colorectal cancer liver metastasis. PLoS One. 2024;19(9):e0307815. PMID: 39259736. doi: 10.1371/journal.pone.0307815.

17. Jin Z, Zou Q, Zhou T, Xue T. Preoperative prediction of early recurrence in patients with BRAF mutant colorectal cancer using a intergrated nomogram. Sci Rep. 2024 Oct 25;14(1):25320. PMID: 39455810. doi: 10.1038/s41598-024-77256-2.

18. Fu S, Chen D, Zhang Y, Yu X, Han L, Yu J, et al. A CT-based radiomics tumor quality and quantity model to predict early recurrence after radical surgery for colorectal liver metastases. Clin Transl Oncol. 2025 Mar;27(3):1198-210. PMID: 39153176. doi: 10.1007/s12094-024-03645-8.

19. Sluckin TC, Hekhuis M, Kol SQ, Nederend J, Horsthuis K, Beets-Tan RGH, et al. A deep learning framework with explainability for the prediction of lateral locoregional recurrences in rectal cancer patients with suspicious lateral lymph nodes. Diagnostics (Basel). 2023 Sep 29;13(19). PMID: 37835842. doi: 10.3390/diagnostics13193099.

20. Hu H, Chi JC, Zhai B, Guo JH. CT-based radiomics analysis to predict local progression of recurrent colorectal liver metastases after microwave ablation. Medicine (Baltimore). 2023 Dec 29;102(52):e36586. PMID: 38206750. doi: 10.1097/md.0000000000036586.

21. Liu Z, Wang Y, Shen F, Zhang Z, Gong J, Fu C, et al. Radiomics based on readout-segmented echo-planar imaging (RS-EPI) diffusion-weighted imaging (DWI) for prognostic risk stratification of patients with rectal cancer: a two-centre, machine learning study using the framework of predictive, preventive, and personalized medicine. Epma j. 2022 Dec;13(4):633-47. PMID: 36505889. doi: 10.1007/s13167-022-00303-3.

22. Jayaprakasam VS, Paroder V, Gibbs P, Bajwa R, Gangai N, Sosa RE, et al. MRI radiomics features of mesorectal fat can predict response to neoadjuvant chemoradiation therapy and tumor recurrence in patients with locally advanced rectal cancer. Eur Radiol. 2022 Feb;32(2):971-80. PMID: 34327580. doi: 10.1007/s00330-021-08144-w.

23. Huang YC, Tsai YS, Li CI, Chan RH, Yeh YM, Chen PC, et al. Adjusted CT image-based radiomic features combined with immune genomic expression achieve accurate prognostic classification and identification of therapeutic targets in stage III colorectal cancer. cancers (Basel). 2022 Apr 8;14(8). PMID: 35454802. doi: 10.3390/cancers14081895.

24. Badic B, Da-Ano R, Poirot K, Jaouen V, Magnin B, Gagnière J, et al. Prediction of recurrence after surgery in colorectal cancer patients using radiomics from diagnostic contrast-enhanced computed tomography: a two-center study. Eur Radiol. 2022 Jan;32(1):405-14. PMID: 34170367. doi: 10.1007/s00330-021-08104-4.

25. Fan S, Cui X, Liu C, Li X, Zheng L, Song Q, et al. CT-Based Radiomics Signature: A potential biomarker for predicting postoperative recurrence risk in stage II colorectal cancer. Front Oncol. 2021;11:644933. PMID: 33816297. doi: 10.3389/fonc.2021.644933.

26. Chen F, Ma X, Li S, Li Z, Jia Y, Xia Y, et al. MRI-based radiomics of rectal cancer: assessment of the local recurrence at the site of anastomosis. Acad Radiol. 2021 Nov;28 Suppl 1:S87-s94. PMID: 33162318. doi: 10.1016/j.acra.2020.09.024.

27. Dai W, Mo S, Han L, Xiang W, Li M, Wang R, et al. Prognostic and predictive value of radiomics signatures in stage I-III colon cancer. Clin Transl Med. 2020 Jan;10(1):288-93. PMID: 32508036. doi: 10.1002/ctm2.31.

28. Jeon SH, Song C, Chie EK, Kim B, Kim YH, Chang W, et al. Delta-radiomics signature predicts treatment outcomes after preoperative chemoradiotherapy and surgery in rectal cancer. Radiat Oncol. 2019 Mar 12;14(1):43. PMID: 30866965. doi: 10.1186/s13014-019-1246-8.
