# Supplementary figures and images for: Predictive Performance of Radiomics-Based Machine Learning for Colorectal Cancer Recurrence Risk: Systematic Review and Meta-Analysis
Source: JMIR Med Inform. 2025 Nov 28;13:e78644. doi: 10.2196/78644 (PMC12669921; doi:10.2196/78644)

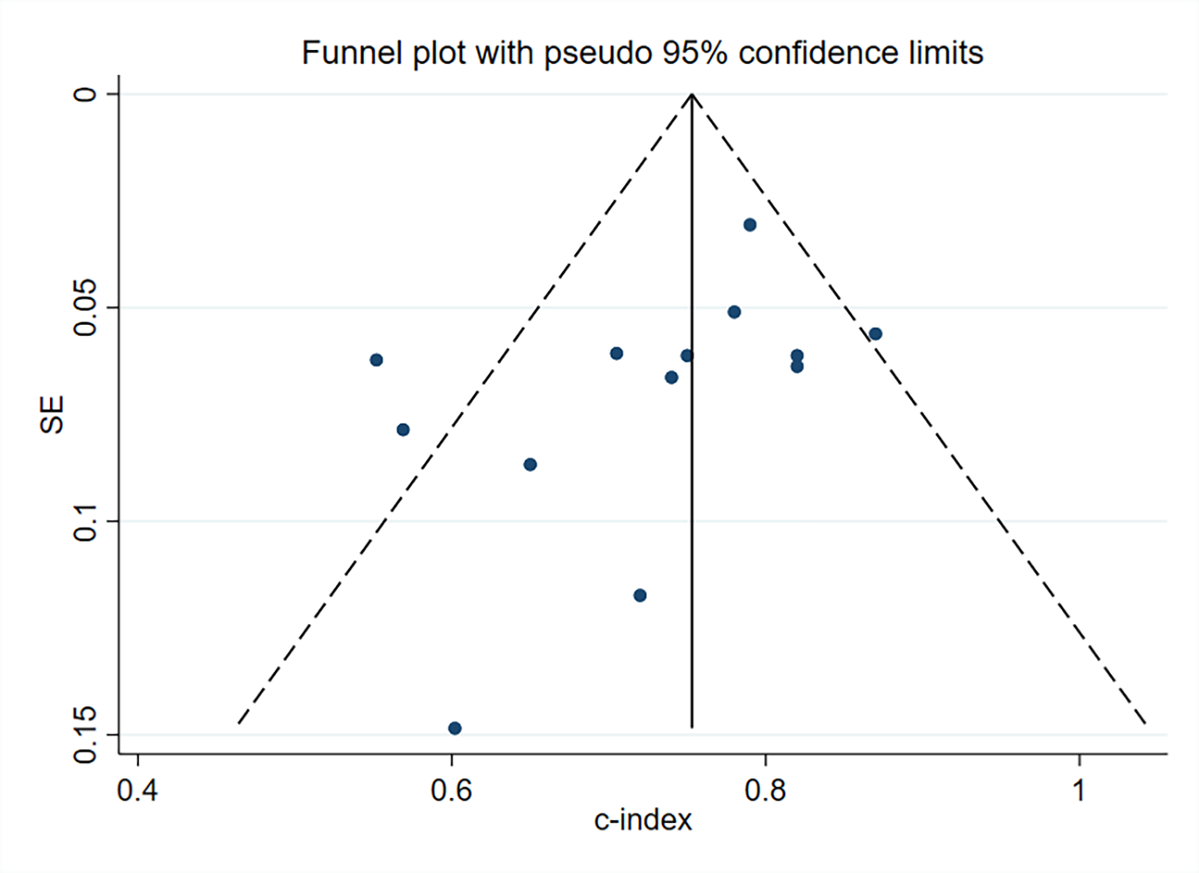

Supplement: Multimedia Appendix 2 [file medinform-v13-e78644-s002.png]

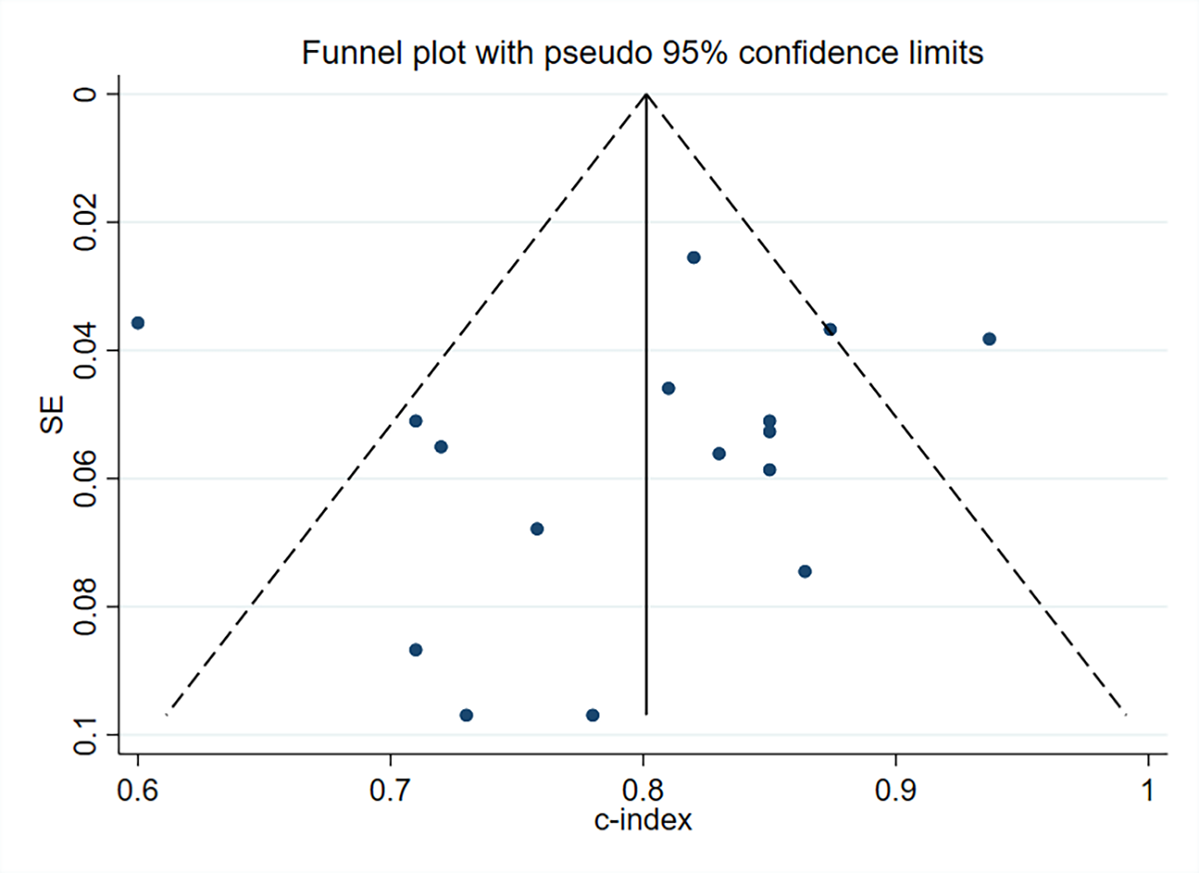

Supplement: Multimedia Appendix 3 [file medinform-v13-e78644-s003.png]

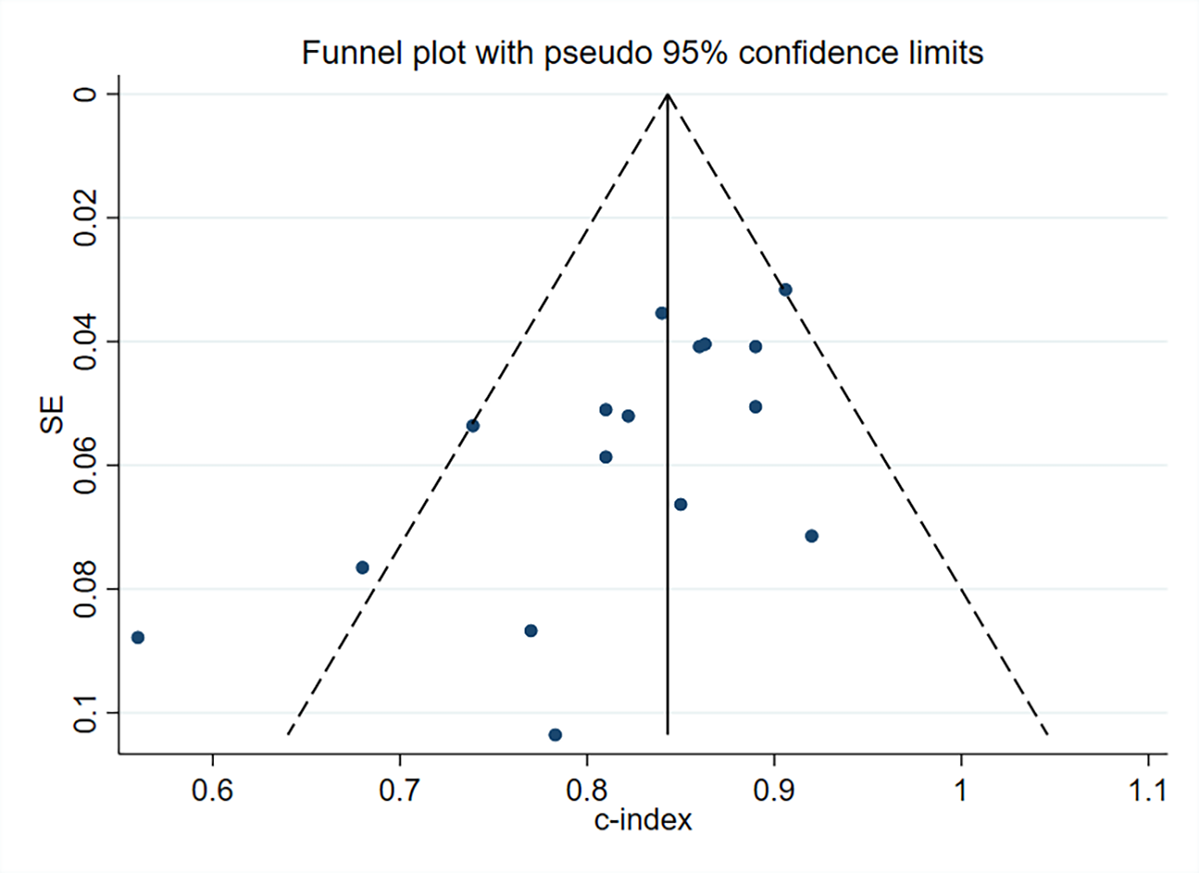

Supplement: Multimedia Appendix 4 [file medinform-v13-e78644-s004.png]

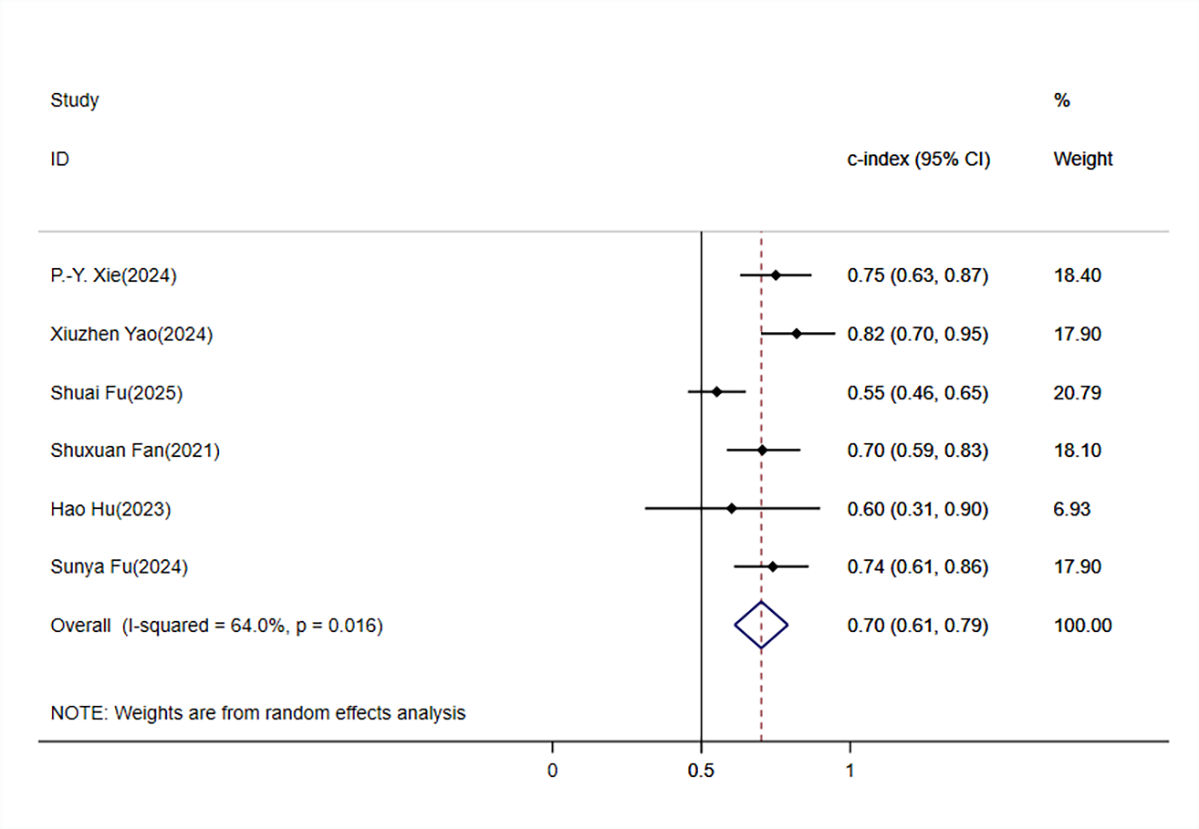

Supplement: Multimedia Appendix 5 [file medinform-v13-e78644-s005.png]

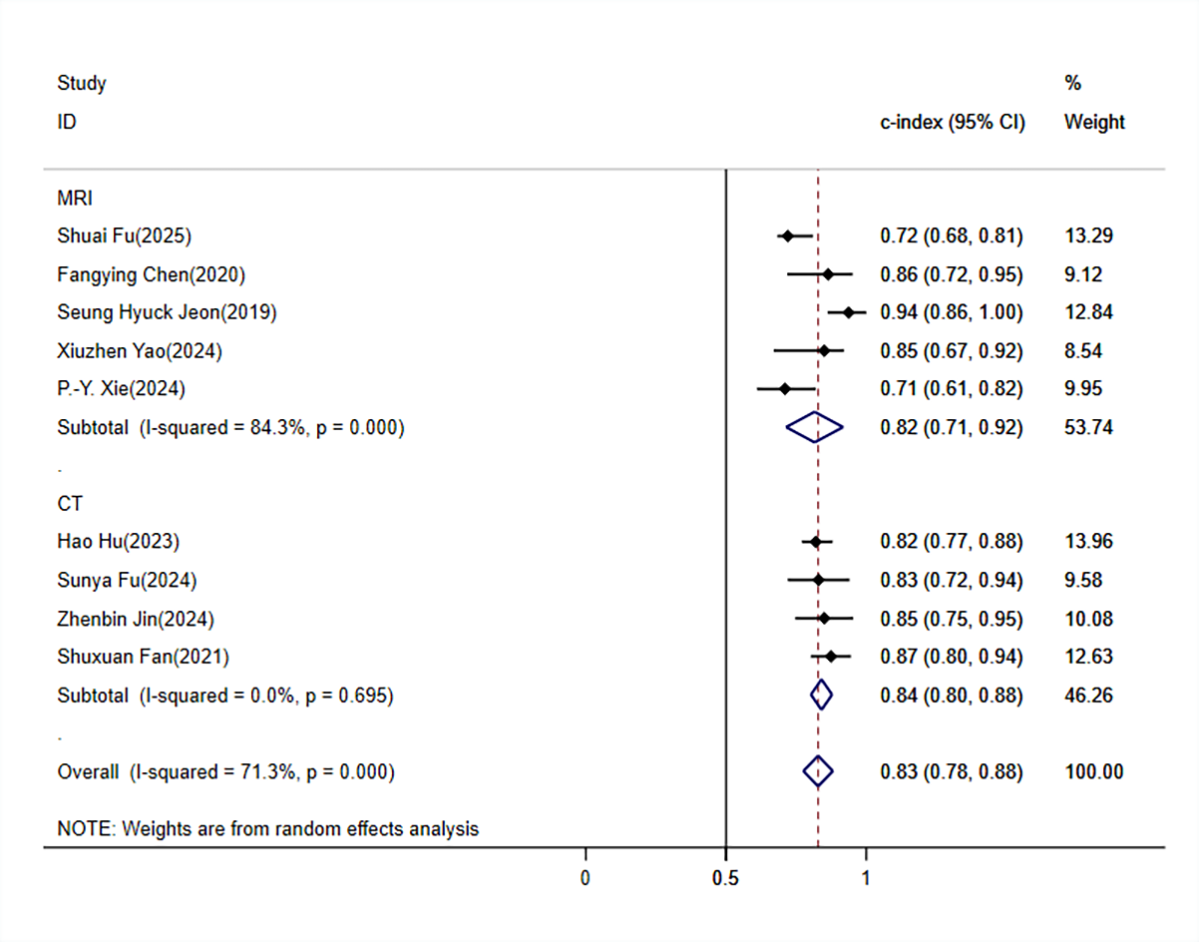

Supplement: Multimedia Appendix 6 [file medinform-v13-e78644-s006.png]

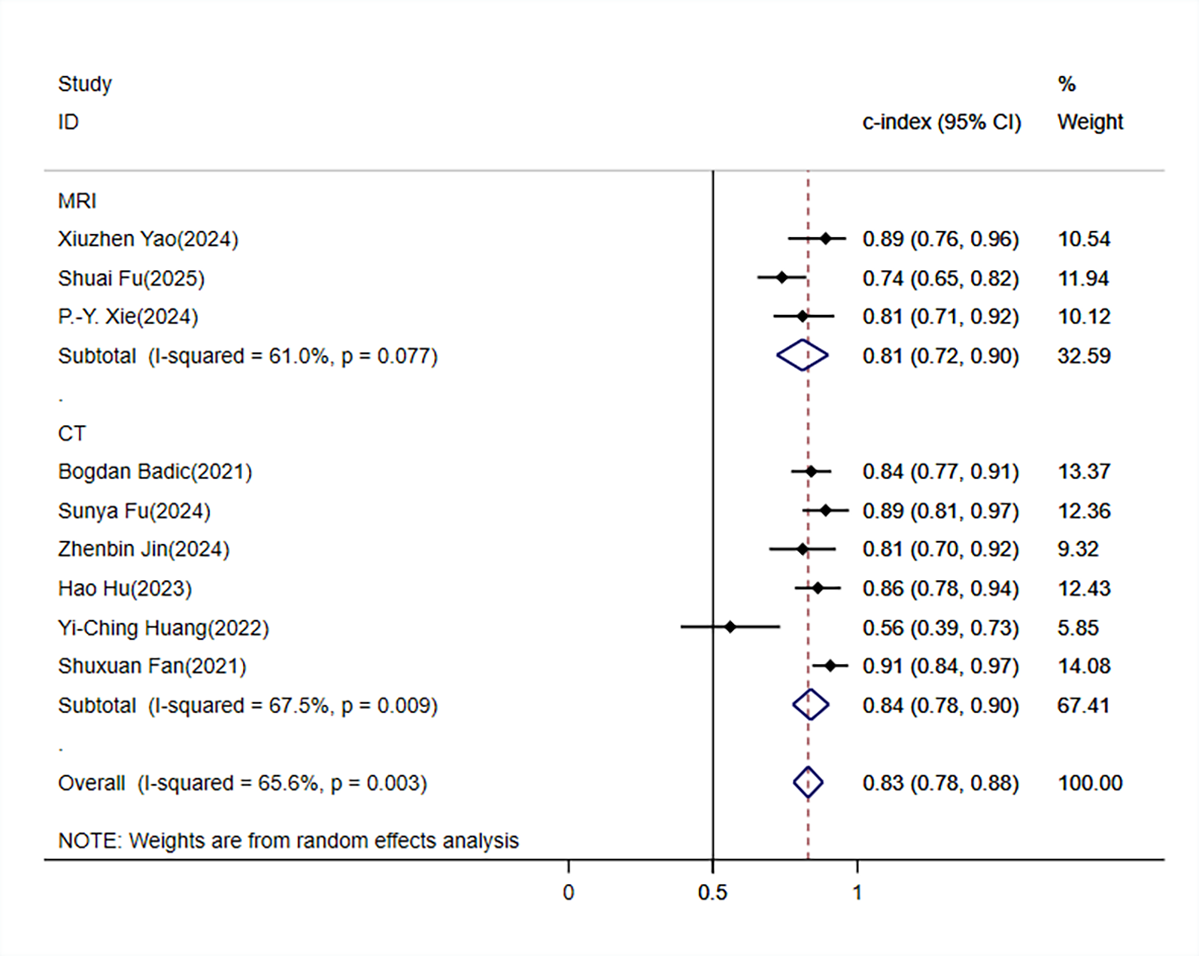

Supplement: Multimedia Appendix 7 [file medinform-v13-e78644-s007.png]

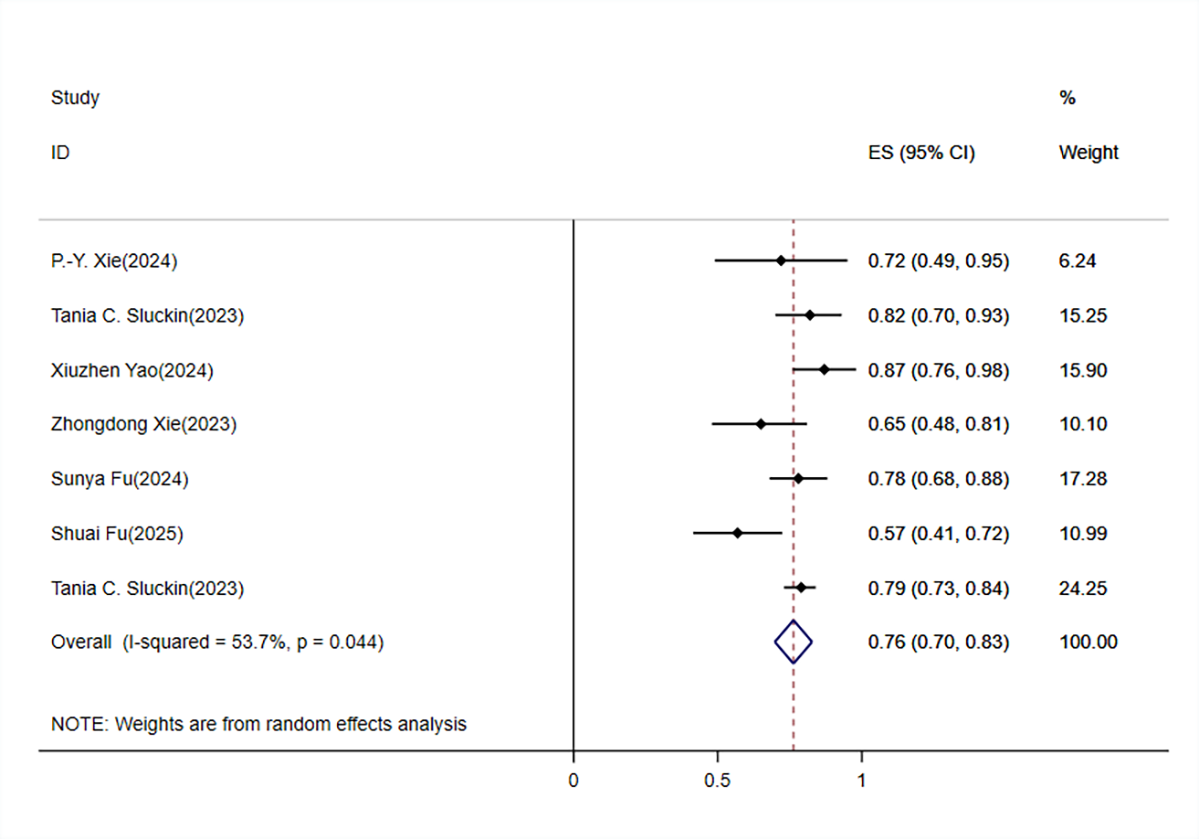

Supplement: Multimedia Appendix 8 [file medinform-v13-e78644-s008.png]

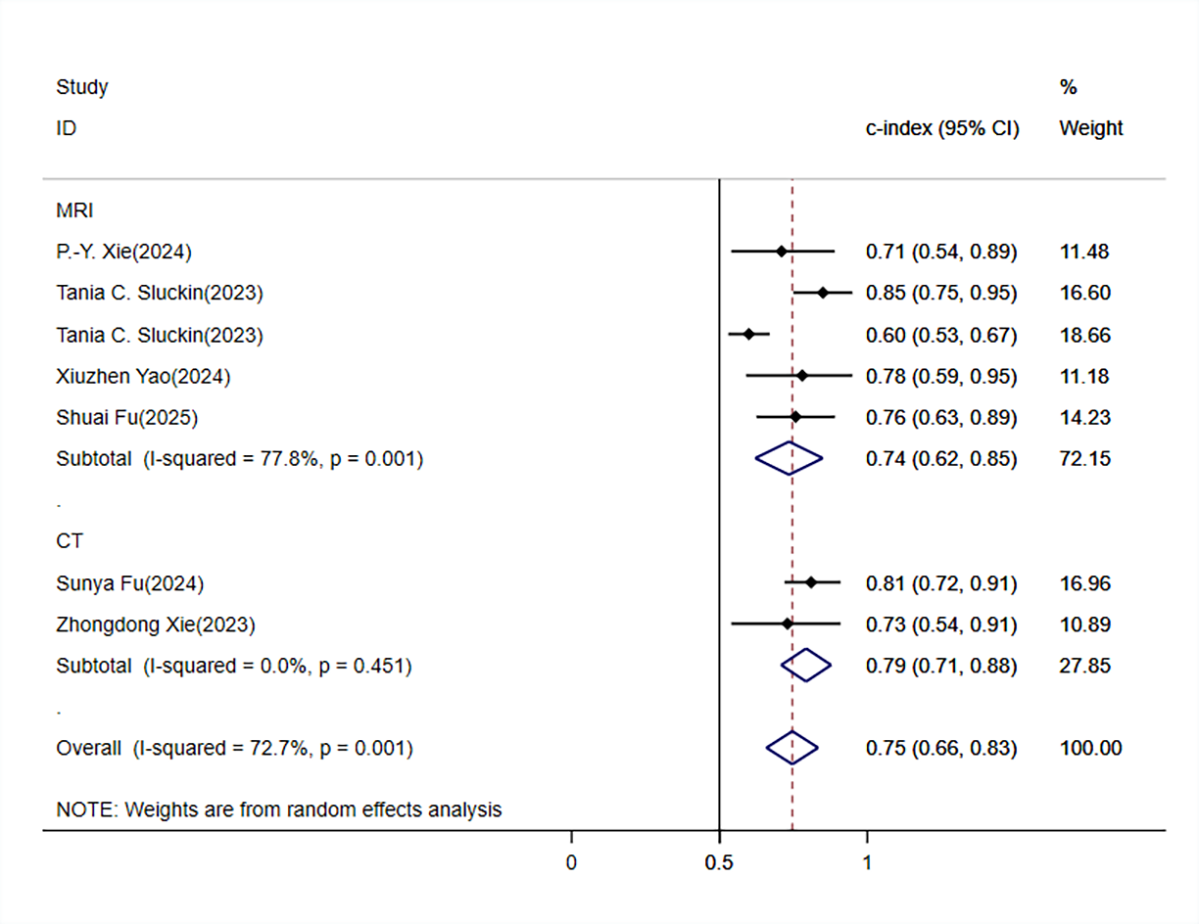

Supplement: Multimedia Appendix 9 [file medinform-v13-e78644-s009.png]

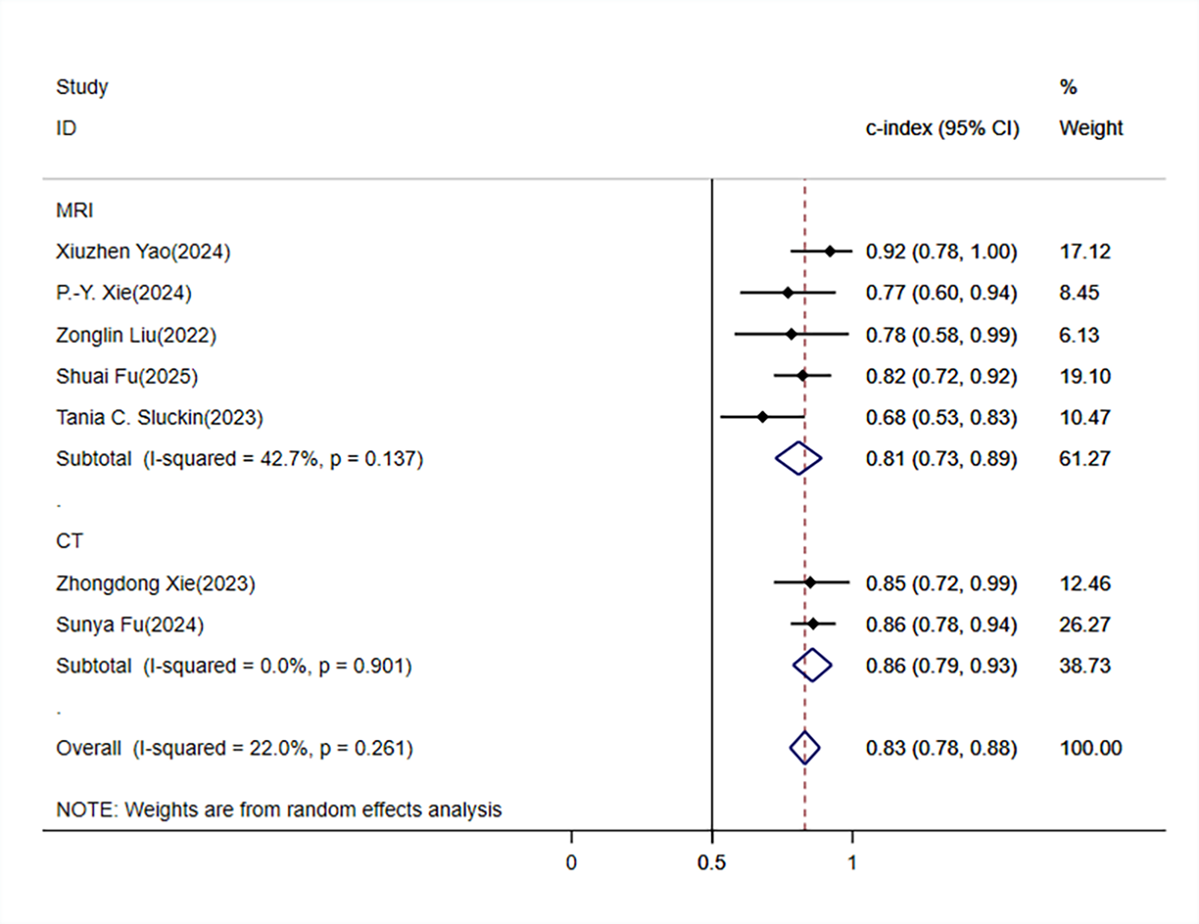

Supplement: Multimedia Appendix 10 [file medinform-v13-e78644-s010.png]
